# Csm4, in Collaboration with Ndj1, Mediates Telomere-Led Chromosome Dynamics and Recombination during Yeast Meiosis

**DOI:** 10.1371/journal.pgen.1000188

**Published:** 2008-09-26

**Authors:** Jennifer J. Wanat, Keun P. Kim, Romain Koszul, Sarah Zanders, Beth Weiner, Nancy Kleckner, Eric Alani

**Affiliations:** 1 Department of Molecular Biology and Genetics, Cornell University, Ithaca, New York, United States of America; 2 Department of Molecular and Cellular Biology, Harvard University, Cambridge, Massachusetts, United States of America; National Cancer Institute, United States of America

## Abstract

Chromosome movements are a general feature of mid-prophase of meiosis. In budding yeast, meiotic chromosomes exhibit dynamic movements, led by nuclear envelope (NE)-associated telomeres, throughout the zygotene and pachytene stages. Zygotene motion underlies the global tendency for colocalization of NE-associated chromosome ends in a “bouquet.” In this study, we identify Csm4 as a new molecular participant in these processes and show that, unlike the two previously identified components, Ndj1 and Mps3, Csm4 is not required for meiosis-specific telomere/NE association. Instead, it acts to couple telomere/NE ensembles to a force generation mechanism. Mutants lacking Csm4 and/or Ndj1 display the following closely related phenotypes: (i) elevated crossover (CO) frequencies and decreased CO interference without abrogation of normal pathways; (ii) delayed progression of recombination, and recombination-coupled chromosome morphogenesis, with resulting delays in the MI division; and (iii) nondisjunction of homologs at the MI division for some reason other than absence of (the obligatory) CO(s). The recombination effects are discussed in the context of a model where the underlying defect is chromosome movement, the absence of which results in persistence of inappropriate chromosome relationships that, in turn, results in the observed mutant phenotypes.

## Author Summary

In meiosis, cells specified to become gametes (eggs or sperm) undergo a single round of DNA replication followed by two consecutive chromosomal divisions. In most organisms, the proper segregation of chromosomes at the first meiotic division is mechanically dependent upon genetic exchange, or crossing over, at homologous sites along chromosomes. This process is highly regulated so that every pair of matched chromosomes, regardless of size, receives at least one crossover. In humans, defects in this recombination process can lead to a variety of chromosome aneuploidy syndromes. During early stages in meiosis, the ends of chromosomes, called telomeres, associate with the envelope of the nucleus and undergo highly dynamic movements. We identified a new component of the movement-generating system, Csm4, in budding yeast. In the absence of Csm4, the telomeres associate with the nuclear envelope but are locked in an immobile state. In addition, strains lacking Csm4 show delayed recombination progression and high levels of chromosome mis-segregation at the first meiotic division. These findings suggest that, during meiosis, Csm4 is involved in coupling telomere complexes to the movement-generating system and that chromosome motion is important for the completion of early steps in recombination.

## Introduction

Classical cytological studies have shown that during the zygotene stage of meiosis, chromosome ends are tightly and specifically associated with the nuclear envelope (NE) and move coordinately into a “bouquet” configuration such that they are localized within a sub-area of the nuclear periphery. Upon exit from this stage, during early pachytene, telomeres again redistribute throughout the nuclear periphery (reviewed in [1]; [2]–[4]). In budding yeast, these global effects are achieved by means of highly dynamic, actin-dependent, telomere-led movements, which, after initiating at the onset of zygotene, continue into pachytene [2],[5],[6]. Recent work from one of our laboratories shows that telomeres and associated nuclear envelope (NE) segments move via passive association with nucleus-hugging segments of dynamic cytoskeletal actin cables that tend to form in the vicinity of the spindle pole body (SPB; [6]). A different mechanism has been elucidated for fission yeast; telomeres are tightly and specifically associated with the SPB and the entire complex moves dynamically along microtubules via interaction with the dynein motor complex [7].

Studies in fission yeast, budding yeast (references below), rat, and mouse [8] have shown that, in accord with their special functions, telomeres of meiotic chromosomes become robustly associated with the NE in complexes comprised of both meiosis-specific proteins and proteins recruited from the mitotic program. In *S. pombe* meiosis, Bqt1 and Bqt2 connect the telomere binding protein Rap1, which associates with telomeres through interactions with Taz1, to the spindle pole body protein Sad1. Sad1 is a member of the SUN domain family of proteins that localize to the NE [3],[9]. Sad1 is also known to interact with the spindle pole body binding (SPB) protein Kms1 [10]. The final telomere/SPB cluster is thought to form through interactions between Bqt1, Bqt2, Rap1, Taz1, Sad1, and Kms1 [3],[7]. In budding yeast, two components of meiotic telomere-NE ensembles have been identified thus far: Ndj1, also called Tam1 [11],[12], and Mps3 [13]. Ndj1 is a meiosis-specific protein that mediates association of telomeres to the NE; as a result, in the absence of Ndj1, global and dynamic chromosome movements are severely reduced ([2],[11],[12],[14],[15]; this work). Mps3, which is present in mitotic as well as meiotic cells, most likely has two roles [13]. First, it interacts directly with Ndj1 such that the two proteins display a mutually dependent requirement for telomere localization to the NE. Second, Mps3 is a SUN domain protein, which suggests that it may mediate interactions between telomeres and cytoskeletal determinants. Recent studies have shown that rapid movement of yeast pachytene chromosomes involves passive association of telomere/NE ensembles to dynamically moving actin cables. Within this mechanically integrated complex, force is exerted on the NE component and transduced via telomere/NE complexes through the NE to the associated chromosome end [6].

The functional role(s) of global and dynamic chromosome movements for meiosis, though widely discussed, are not established. In both fission yeast and budding yeast, situations in which telomere localization is aberrant or the motion mechanism is directly abrogated reveal diverse defects. In *S. cerevisiae*, *ndj1Δ* strains show levels of crossing over similar to wild-type (WT), partially disrupted crossover (CO) interference, modestly increased ectopic recombination, delayed formation of tight juxtaposition of homologs including delayed formation of the synaptonemal complex (SC), defective progression of recombination intermediates into mature recombinants, increased MI nondisjunction, and decreased spore viability [11], [12], [16]–[18]. In *S. pombe*, similar phenotypes are observed for mutants defective in telomere localization; however, in contrast to budding yeast, CO-levels are significantly decreased (e.g. [3],[19]). Furthermore, in this organism, meiosis does not involve CO interference or the SC, and it has been difficult to perform a detailed analysis of recombination intermediates and their timing. These findings have led to suggestions that motion might play a direct role in recombination and/or homolog juxtaposition. However, the pleiotropic nature of these effects have made it difficult to distinguish defects that are direct consequences of the absence of motion rather than indirect effects and/or those that result from aberrant telomere biology irrespective of motion. We and others [1],[6],[20],[21] have argued that the primary role of movement is to eliminate aberrant topological relationships among chromosomes, e.g. entanglements or “interlocks” and/or other types of unprogrammed connections among nonhomologous chromosomes, a possibility that has not yet been directly assessed in any organism.

The present study began with a search for mutations that affect recombination through telomere-dependent effects. The hallmark phenotype conferred by the *ndj1Δ* mutation is increased nondisjunction of homologs at the MI division, and a screen for mutants defective in chromosome segregation during meiosis [22] identified three additional genes with weak chromosome missegregation phenotypes. We began by further characterizing these missegregation phenotypes. We show that one of these genes, *CSM4*, is required specifically for regular segregation of homologs, analogously to *NDJ1*, and by several additional criteria, encodes a third participant in the meiotic telomere/NE interactions involved in motion. We further show that the role of Csm4 is distinct from that of either Ndj1 or Mps3. Finally, we analyzed diverse *csm4Δ* and *ndj1Δ* phenotypes for motion at zygotene and pachytene, recombination (by genetic and physical approaches), SC morphogenesis, and meiotic progression. The observed phenotypes suggest a role for chromosome motion that can explain all observed effects and also supports the idea that the primary role of motion is regularization of topological relationships among chromosomes. Related and complementary findings are presented in the accompanying paper by Shinohara and colleagues ([23], see also [24]).

## Results

### Analysis of Chromosome Mis-Segregation

Ndj1 (also called Tam1) was the first identified component of yeast telomere/NE ensembles [11],^12^. The hallmark phenotype of *ndj1Δ* is nondisjunction of homologs at the first meiotic division, with an accompanying modest decrease in spore viability, to 62–82%, as compared to 92–98% in WT [11],[12]. To identify new mutations that affect chromosomal events during meiosis, and in particular recombination, we focused on three genes, *CSM2*, *CSM3*, and *CSM4* (chromosome segregation in meiosis; [22]), whose corresponding mutations confer phenotypes similar to those of *ndj1Δ*: decreased spore viability and aberrant meiotic chromosome segregation.

### MI Homolog Nondisjunction Occurs in *csm4Δ* Mutants

**Figure 1:**
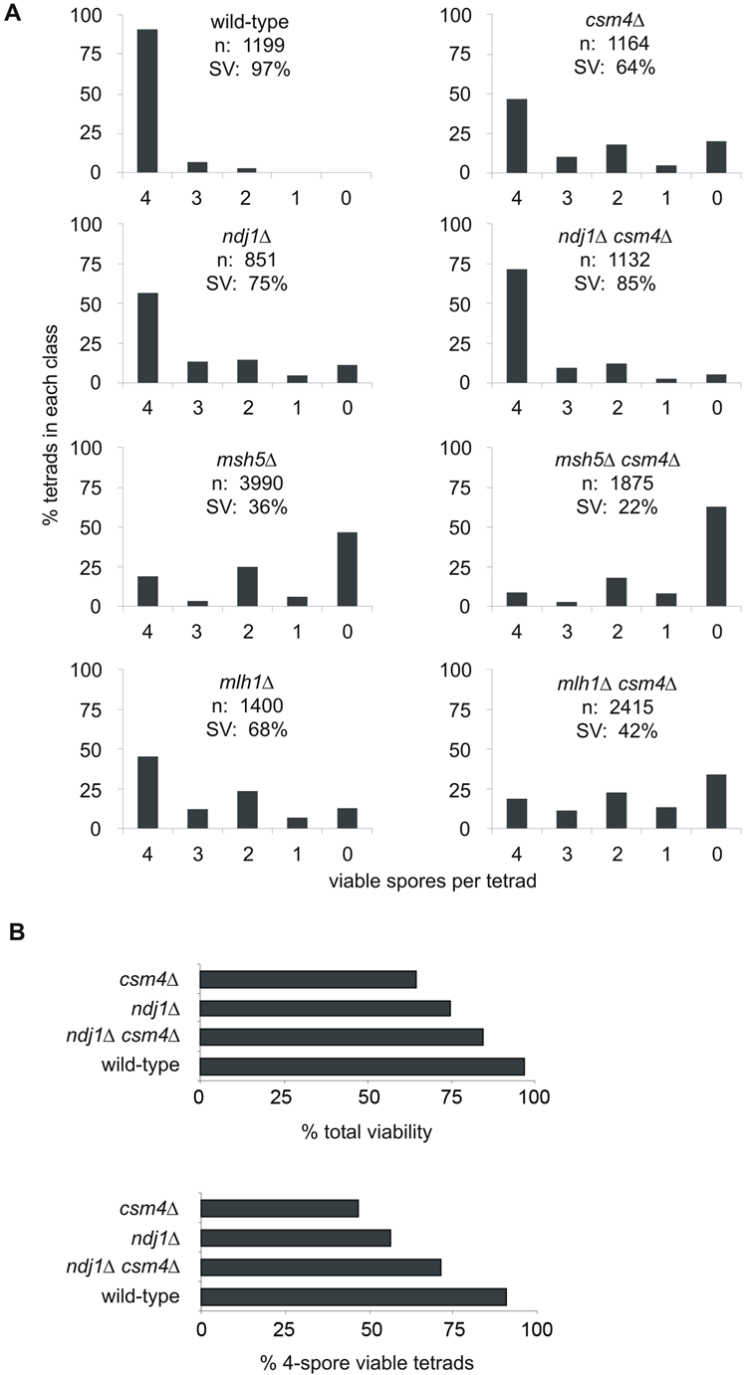
Distribution of viable spores in tetrads dissected from the indicated EAY1108/EAY1112 derived strains. A) In all plots the horizontal axes correspond to the classes of tetrads with 4, 3, 2, 1, and 0 viable spores, and the vertical axes correspond to the percentage of each class. The total number of tetrads dissected (n) and the overall spore viability (SV) are shown for each genotype. B) Histograms representing percent total spore viability and the proportion of four viable tetrads for WT, *csm4Δ*, *ndj1Δ*, and *ndj1Δ csm4Δ* are presented.

We began by further characterizing the nature of the chromosome mis-segregation defect in *csm* mutants. Spore viability patterns revealed that *csm4Δ* confers a pattern that is diagnostic of homolog nondisjunction: an excess of tetrads containing 0, 2, or 4 viable spores as compared to 1 or 3 viable spores [25]. Of the three *csm* mutants, only *csm4Δ* displays this pattern (Figure 1A, data not shown). Csm4 was identified by bioinformatic analysis as a 156 amino acid tail-anchored membrane protein. Consistent with this designation, Csm4 was observed in both the endoplasmic reticulum and the perinuclear membrane when overproduced in mitotic cells [26].

The homolog nondisjunction phenotype of *csm4Δ* was confirmed and extended as follows:

- One approach utilized congenic SK1 strains EAY1108/EAY1112 in which one chromosome (XV; 1040 kb) is heterozygous for the centromere-linked markers, *URA3* and *TRP1* ([27], Table S1). In this background, analysis of asci containing two viable spores can distinguish a MI segregation defect from random spore death. In the former case, the two spores often contain the same (sister) centromeric marker while, in the latter case, there is no such tendency (e.g. [27],[28]). Consistent with an MI defect, 88% of two-spore viable *csm4Δ* tetrads (n = 212) contained sister centromere markers.
- In the absence of any other defect, nondisjunction of the centromere-marked chromosome XV homologs will result in two-spore viable tetrads in which each spore contains one centromere from each homolog; as a result, both spore clones will be Ura^+^ Trp^+^. In *csm4Δ* 1.4 % of the two-spore viable tetrads were of this type (n = 212). This level of homolog nondisjunction is very similar to that observed in the analysis of two recombination mutants known to confer homolog nondisjunction: *mlh1Δ* (1.2%, n = 324) and *msh5Δ* (3.4%, n = 994).
- Analogously, nondisjunction of the unmarked chromosome III of these strains will yield two-spore viable tetrads in which the two spores carry both yeast mating types (*MAT*a and *MATα*). Such spores can be detected because they fail to mate with haploid tester strains of either mating type. In *csm4Δ*, 7.8% (n = 103) were of this type, with a similar level observed for *msh5Δ* (7.1%, n = 56).
- In contrast, another common type of MI missegregation, precocious segregation of sister chromatids (PSSC), occurs at a very low level in *csm4Δ*. PSSC can be identified by analyzing two- and three-spore viable tetrads [29]. In the EAY1108/EAY1112 strain set, PSSC was not detectable in either WT or *csm4Δ*; in another background (SK1 isogenic NH942/NH943; Table S1) *csm4Δ* strains showed 5 PSSC events in 1284 tetrads (0.39%) and WT showed no PSSC events in 646 WT tetrads (difference between WT and *csm4Δ* values is not statistically significant, Fisher's Exact Test).

Taken together, these analyses show that the primary segregation defect in *csm4Δ* mutants is homolog nondisjunction. Similar results are reported by Kosaka et al. [23].

### Csm4 and Ndj1 Play Functionally Related Roles for Homolog Disjunction

Comparison of isogenic strains reveals that the phenotype of *csm4Δ* is significantly stronger than that of *ndj1Δ* (Figure 1A). As previously mentioned, *csm4Δ* mutants display a spore viability pattern indicative of nondisjunction (4, 2, 0>3, 1 viable spores). This pattern is more severe in *csm4Δ* vs. *ndj1Δ* (Figure 1B). Further, a larger percentage of two-spore viable tetrads are sisters in *csm4Δ* (88%) compared to *ndj1Δ* (69%). As judged by the first two of these phenotypes, the double mutant defect is very similar to *ndj1Δ*, but slightly weaker (Figure 1). These same patterns are also apparent in the percentage of four viable spore asci and overall spore viability (Figure 1A and B). These mutant phenotypes imply functional interaction between Csm4 and Ndj1 with respect to MI homolog disjunction. The observed epistatic relationship is intriguing. First, it is the *weaker* phenotype that dominates (is epistatic to) the *stronger* phenotype. Second, the occurrence of slight synergy implies that not only is Ndj1 strongly required for the *csm4Δ* phenotype but conversely, Csm4 is weakly required for the *ndj1Δ* phenotype. Importantly, the above conclusions do not reflect differences in sporulation efficiencies. For WT, 82% of cells yielded asci with three or four spores and 89% yielded asci with at least one spore (n = 234 cells examined); *csm4Δ*, *ndj1Δ*, and *csm4Δ ndj1Δ* all exhibited similar reductions in both categories: 50, 51, and 64% respectively, and 73, 59, and 78% respectively (n = 229, 221, and 238 cells examined, respectively).

### Telomere/NE Analysis

**Figure 2:**
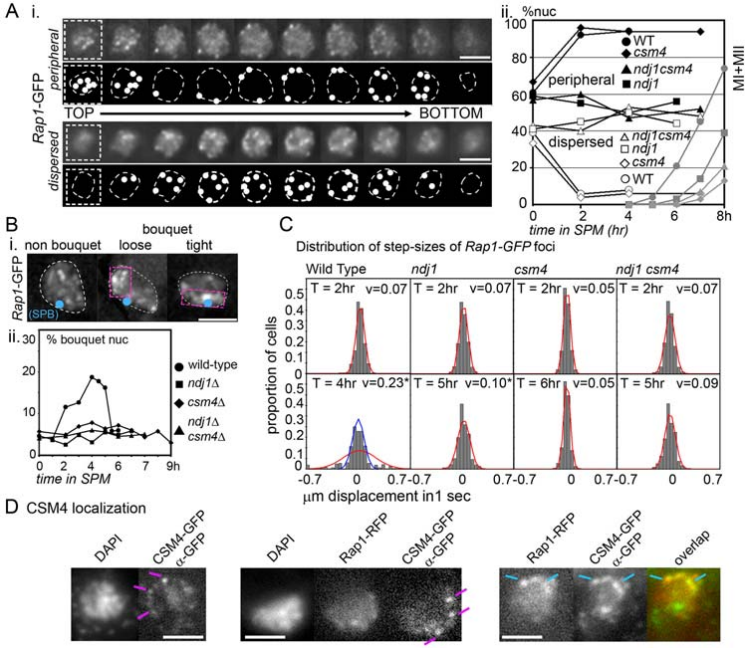
Telomere organization and dynamics in WT, *ndj1Δ*, and *csm4Δ* mutants. A) Telomere organization in strains whose telomeres are illuminated by Rap1-GFP. i Telomere organization in fixed prophase nuclei. Nuclei were distributed into two classes, “peripheral” (top panel; 10 frames from top to bottom) and “dispersed” (bottom panel) depending on the absence or presence of distinguishable Rap1-GFP foci within the nuclear volume, respectively (Materials and Methods). ii The proportions of these two categories (left Y axis) in WT (NKY4000) and *ndj1Δ*, *csm4Δ,* and *ndj1Δ csm4Δ* backgrounds (NKY3906, NKY3904, and NKY3905, respectively) were plotted as a function of time in sporulation media (SPM), with full and empty symbols on black lines corresponding to peripheral and dispersed proportions, respectively (Note: the differences between the two categories, while not absolute, is sufficiently robust that an untrained observer can place nearly all nuclei (>90%) in one category or the other without ambiguity. This robustness is further supported by the regular progression of changes in disposition over time not only in WT but also in mutants (below). The right Y axis and gray lines indicate the percentage of cells that have completed at least MI. B) Analysis of “bouquet” distribution in fixed nuclei. i 2D projections from 3D acquisition of Rap1-GFP signals, with the position of the SPB indicated by a blue circle (Materials and Methods). The nuclei presented here display peripherally localized telomere signals with either no evidence of bouquet colocalization or with “loose” and “tight” colocalization in the vicinity of the SPB. The region where most Rap1 foci co-localize is framed by a pink dotted rectangle. White dotted line indicates the outline of the nucleus, as estimated from Rap1-GFP background. Complete sets of 3D Rap1-GFP images and definitions of the three categories are provided in Figure S1. ii Proportion over time of nuclei exhibiting a “bouquet” configuration in WT, *ndj1Δ*, *csm4Δ,* and *ndj1Δ csm4Δ* backgrounds (NKY4000, NKY3906, NKY3904, and NKY3905, respectively; ∼100 nuclei per timepoint). C) Chromosome motion during prophase. i For statistical convenience, the histograms of the x and y step-sizes of Rap1-GFP foci, recorded every second over 1 min, were plotted prior to zygotene (t = 2 h for WT and *ndj1Δ/csm4Δ* mutants, respectively), and during zygotene stages (t = 4, 5, 6, and 5 h for WT, *ndj1Δ*, *csm4Δ*, and *ndj1Δ csm4Δ* backgrounds, respectively; Materials and Methods). Red curves indicate the distribution expected when assuming that the displacements follow a Normal distribution. For WT t = 4 h, the blue curve represent the expected Normal distribution of the step sizes included within v (t = 2 h)±2 S.D. (i.e. 77% of total measurements). v indicates the mean velocity of telomeric 2D displacements over time. An accompanying asterisk indicates a statistically significant difference in velocity the between pre-zygotene and zygotene stages (t-test, 0.05 significance level; Material and Methods). D) Csm4 localization in WT cells expressing Rap1-RFP and GFP-Csm4 (EAY1797). Fixed cells were spread and hybridized with anti-GFP antibodies. Pink and turquoise lines outline the nuclear peripheral Csm4 signal and co-localization of Rap1 and Csm4 signals, respectively. All scale bars represent 2 µm.

Association of telomeres with the NE, occurrence of the bouquet configuration, and dynamic telomere movements were assessed, in SK1 isogenic strains (Table S1) by analyzing the disposition of Rap1-GFP foci. Rap1 localizes prominently and focally at telomeres and less markedly throughout chromatin (Figure 2A, panel i; [5]). Our approach can detect foci that correspond to single bivalent telomeres at pachytene (R. Koszul, unpublished data), and thus, for earlier stages, should be sensitive enough to detect clusters of two (or more) unpaired homolog telomeres or four (or more) individual chromatids. Since bouquet formation involves colocalization of telomeres near, but not at, the SPB (Introduction), we used strains in which the SPB was also labeled, with Spc42-RFP (Figure 2B, panel i; Figure S1; Table S1; Materials and Methods).

Cells were taken through synchronous meiosis under standard conditions (Material and Methods). In such cultures, at any given time point, the majority of nuclei are in one particular stage. Specifically, at t = 2, 3, 4 and 5 h, the majority of nuclei are in G2, leptotene, zygotene, and pachytene respectively, as defined by fluorescence activated cell sorter (FACS) analysis and SC status (e.g. [6] and below). Zygotene and pachytene nuclei can thus be defined operationally by the population average behavior of nuclei at t = 4 and t = 5 h, respectively, albeit with some “contamination” from other stages at each time point.

### WT Meiosis

Organization of Rap1-GFP foci was analyzed in nuclei of living cells by 3D acquisition, in which a series of 400 nm optical z-sections are taken over time (Figure 2A; n = 50 cells at every time point; 10 planes total, exposure time of 900 ms; Material and Methods). In WT mitotic cells (t = 0 h in SPM), nuclei could be sorted by visual inspection into two categories (Figure 2A, panel i, panel ii, t = 0): ∼60% showed a ring of Rap1-GFP foci located in the periphery of the main chromosomal mass, with no clearly discernable internal foci, implying that telomeres are located “peripherally”. The remaining ∼40% clearly showed internal foci, implying a “dispersed” disposition pattern. Similar categories have been seen in other studies (e.g. [15]). In contrast, by 2 h after initiation of meiosis, most nuclei were in the peripheral configuration (Figure 2A, panel ii). This progression presumably reflects complete migration of telomeres to the nuclear periphery via formation of meiosis-specific telomere/NE complexes that have assembled in early prophase. Since meiotic telomere/NE association at G2/leptotene is a regular feature of meiosis in many organisms (e.g. [30],[31]), we infer that yeast exhibits this same progression but with a prior “background” from mitotic telomere/NE association.

Living cells were also analyzed for the movement of Rap1-GFP foci. For this purpose, the focal plane of the microscope was set at the top of each examined nucleus so that movements around the nuclear periphery could be observed in apparent two dimensions. Frames were taken at one-second intervals over a period of one minute. The positions of the spots present in such focal planes were recorded and analyzed using SpotTracker2D ImageJ plug-in [32], when the amplitude of the displacement was limited, or manually at t = 4 h (below). Such analysis was performed for 5–12 Rap1 foci taken from a minimum of 5 different nuclei (yielding a total of 340 one-second step-sizes for both time points). At t = 2 h, when telomeres have reached their peripheral localization, the average velocity of movement (v) through two-dimensional space was 0.07±0.05 (S.D.) µm/sec. Further, these step sizes exhibit nearly (but not perfectly) a Gaussian distribution, suggesting that all foci are behaving similarly (Figure 2C, red curve; for details see figure legend and Materials and Methods). The same features are also seen previously at t = 0 h (average velocity 0.06±0.05 µm/sec with a near-Gaussian step-size distribution), in accord with the fact that active motion has not yet begun by t = 2 h [6]. In contrast, at t = 4 h, foci exhibit an increased average velocity of 0.23±0.23 µm/sec, in accord with earlier studies [5],[6]. Moreover step-sizes no longer fit a Gaussian distribution (Figure 2C, red curve); instead, there appear to be two types of movement, with a majority of steps being smaller and corresponding to a near-Gaussian distribution (Figure 2C, blue curve; ∼77% of total) plus a minority of much larger steps. The two apparent subpopulations exhibit velocities of ∼0.1 and ∼0.45 µm/sec, respectively, both of which are greater than the velocity observed at t = 2 h (∼0.07 µm/sec). The existence of two such populations is in good agreement with the fact that, during the period of active actin-mediated movement, only a subset of telomeres are directly coupled to the motion-generating mechanism while others are either unaffected or dragged along passively [2],[6],[24].

To determine the overall disposition of telomeres at various stages, formaldehyde-fixed nuclei were analyzed in 3D by collection of an appropriate set of “z-sections” (Materials and Methods; n = 100 nuclei for each time point). Nuclei in which bright Rap1-GFP foci (i.e. the telomeres) were in a peripheral configuration (above) were scored with respect to whether most of the signals were or were not detectably clustered and, if so, whether those clusters occurred in the vicinity of the SPB (representative examples in Figure 2Bi; details in Figure S1). SPB-associated colocalization was defined as “bouquet”. Such configurations include both “loose bouquet” and “tight bouquet” (Figure 2B, panel i), a distinction previously documented for yeast and several other organisms (e.g. *Sordaria*, D. Zickler, personal communication; [5],[33], Kosaka et al., accompanying paper [23]).

Bouquet nuclei gradually increased in frequency from 2 h after meiosis induction, peaked at t = 4–5 h (i.e. zygotene/pachytene), and then diminished dramatically when cells entered pachytene in accord with expected loss of the bouquet configuration at this stage (Figure 2B, panel ii; analogous results obtained in a second independent experiment, not shown). As also noted in early studies (e.g. Ref. [5],[13],[15],[24]), the proportion of bouquet nuclei is low (∼20%) even at the peak time points. This likely reflects the fact that zygotene nuclei have the potential for telomeres to be in the bouquet configuration but are undergoing such complex dynamic telomere movements that all telomeres are only present in a common area some fraction of the time [5].

### *ndj1Δ/csm4Δ* Mutant Meiosis

*ndj1Δ/csm4Δ* mutants and WT were analyzed for telomere-related events in parallel. All three mutants exhibit a WT mitotic-like configuration at t = 0. However, during meiosis, *ndj1Δ* telomeres fail to progress to a fully peripheral localization pattern (as shown previously; [15]) while, in contrast, *csm4Δ* telomeres behave indistinguishably from WT (Figure 2A, panel ii). Thus, while Ndj1 is required for meiosis-specific telomere/NE association, Csm4 is not, as also shown by Kosaka et al [23]. Further, the *ndj1Δ csm4Δ* double mutant exhibits the *ndj1Δ* phenotype (Figure 2A, panel ii), in accord with previous indications that Ndj1 localizes to telomeres and directly mediates their meiotic NE targeting [15].

*ndj1Δ/csm4Δ* mutants were analyzed for telomere movement at t = 0 and at zygotene, the time of which was defined for all three mutants by analysis of SC formation (below). Differences among different situations were evaluated for significance by comparison of step-size distributions by parametric tests (Materials and Methods). By this criterion, the following patterns emerge: (i) At t = 2 h, all three mutants exhibit velocities of movement similar to that seen in WT. (ii) At zygotene, all three mutants exhibit significantly less movement than WT, implying that Csm4, like Ndj1 [2] is required for active motion (Figure 2C; see also [6],[24]. Since telomeres are still NE-associated in the absence of Csm4, these findings suggest that this molecule is involved in the motion-producing force-generating process *per se*. (iii) Interestingly, from t = 0 to zygotene, there is a small but significant increase in motion in the absence of Ndj1 but no significant change in the absence of Csm4 (Figure 2C). There is also no significant increase when both proteins are absent. This suggests that *csm4Δ* is partially epistatic to *ndj1Δ* with respect to zygotene motion (see Discussion). Other studies further show that Ndj1 is not required for the NE deformations that signal actin-mediated motion while absence of Csm4 completely abrogates such motions [6]. Thus, the residual Csm4-dependent movement observed in *ndj1Δ* appears to reflect residual movement that is independent of meiosis-specific telomere/NE association, e.g. via mitotic-like or non-specific associations. In the absence of Csm4, in contrast, telomeres may simply be “not moving” or may actually be “locked in place.”

In accord with abrogation of telomere/NE association and/or chromosome movement, there is no detectable bouquet formation in *ndjΔ*, *csm4Δ*, or the *ndj1Δ csm4Δ* double mutant (Figure 2B, panel ii). This is also consistent with data reported for *ndj1Δ* [15] and for *csm4Δ* by Kosaka et al. [23].

### Csm4 Co-Localizes with Telomeric Rap1-GFP at the Nuclear Periphery

We also explored the cytological localization of Csm4 during meiosis in relation to the localization of telomeres using a strain (EAY1797) carrying an integrated Csm4-GFP fusion driven from the native *CSM4* promoter and the Rap1-RFP fusion (Materials and Methods). Intrinsic Csm4-GFP fluorescence is sufficiently weak so that localization can only be assessed with anti-GFP antibody in fixed cells, and even then, with substantial background staining. Nonetheless, at mid-prophase, Csm4 can be seen in foci around the periphery of the nucleus (Figure 2D, left). These foci often overlap with strong foci of Rap1-RFP (Figure 2D, right). These images provide evidence suggestive of NE localization of Csm4 and a tendency for association with telomeres. A strain expressing only Rap1-RFP does not show such patterns (data not shown; strain NKY4005).

### Genetic Analysis of Recombination

Defects in MI homolog segregation often reflect defects in the formation of crossovers (COs). Further, it would be interesting to know whether/how telomere dynamics affect recombination. We therefore examined recombination in *csm4Δ* by both genetic (this section) and physical analyses (below).

### Increased Crossing Over in *csm4Δ*

**Figure 3:**
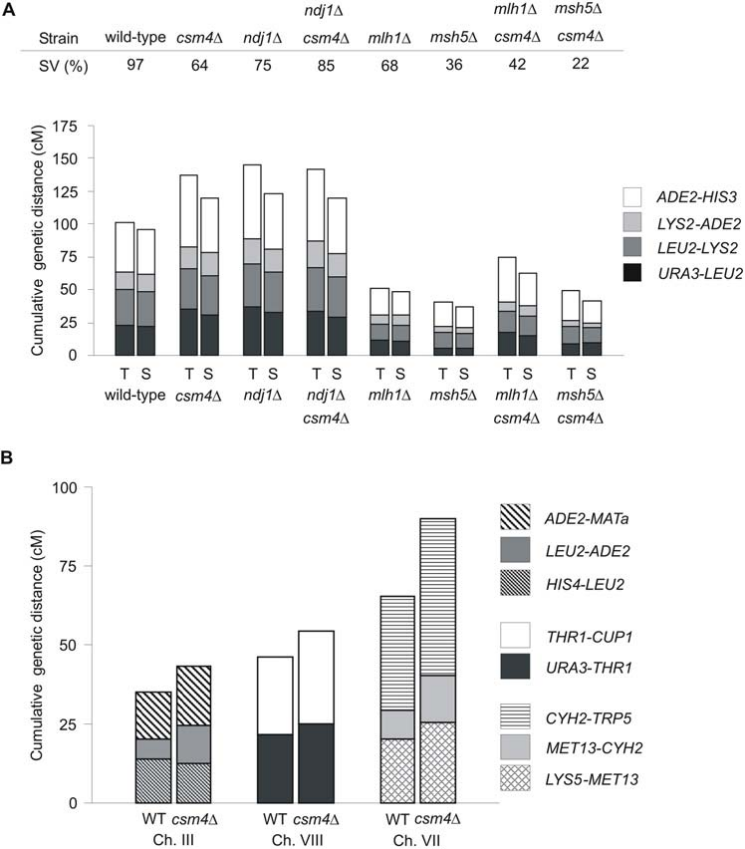
Cumulative genetic distance (cM) in WT, *csm4Δ*, *mlh1Δ*, *msh5Δ*, and *ndj1Δ* strains. In panels A and B, each bar is divided in sectors corresponding to genetic intervals in the region of the chromosome analyzed. A) Cumulative genetic distances between *URA3* and *HIS3* on chromosome XV in EAY1108/EAY1112 derived strains measured from tetrads (T) and single spores (S). B) Cumulative genetic distances between *ADE2* and *LEU2* on chromosome III, *URA3* and *CUP1* on chromosome VIII, and *LYS5* and *TRP5* on chromosome VII in the NH942/NH943 derived strains measured from tetrads (T) and single spores (S). See Tables 1 and S2 for raw data.

We examined crossing over in 12 different intervals by tetrad analysis in WT and *csm4Δ* (Figure 3, Tables 1, S1, and S2). The *csm4Δ* mutation conferred a 30–40% increase in the level of COs for all four intervals in the SK1 congenic strains. In the analysis of complete tetrads, the *URA3-LEU2* and *ADE2-HIS3* intervals were significantly different from WT (G-test, p<0.007, 95% confidence level, Dunn-Sidak correction, [34],[35]) but the *LEU2-LYS2* (p = 0.07) and the *LYS2-ADE2* (p = 0.013) were not (Figure 3A). However, in the spore analysis, only the *LEU2-LYS2* interval (p = 0.014) was not significantly different from WT (p<0.007, Figure 3A). Similarly, in isogenic SK1 strains, CO frequencies were increased in *csm4Δ* mutants at four out of eight analyzed intervals in complete tetrads and at six out of eight intervals in the spore analysis (G-test, p<0.05, 95% confidence level). At the *HIS4-LEU2* interval on chromosome III, CO levels were indistinguishable between WT and *csm4Δ* in both data sets (Figure 3B).

**Table 1:** Genetic map distances (cM) and the distribution of parental and recombinant progeny for the EAY1108/EAY1112 strain background in WT, *csm4Δ*, *ndj1Δ*, *msh5Δ*, and *mlh1Δ* strains on chromosome XV.

Relevant genotype	Tetrads^a^					Single spores^b^			
	Number analyzed	cM	PD	TT	NPD	Number analyzed	cM	Parental	Recombinant
***URA3-LEU2*:**									
wild-type	1068	21.8–23.8	607	456	5	4644	20.6–23.0	3635	1009
*csm4Δ*	531	33.3–36.9	203	319	9	2999	29.3–32.6	2072	927
*ndj1Δ*	472	34.9–39.1	173	289	10	2548	31.0–34.7	1712	836
*mlh1Δ*	616	10.3–12.5	486	128	2	3792	9.6–11.6	3393	399
*msh5Δ*	720	5.0–6.4	643	76	1	5674	5.1–6.3	5352	322
*csm4Δ ndj1Δ*	789	31.8–35.0	337	437	15	3836	27.4–30.3	2732	1104
*csm4Δ mlh1Δ*	418	15.4–19.2	298	115	5	4036	13.6–15.8	3446	590
*csm4Δ msh5Δ*	155	7.5–10.5	127	28	0	1624	8.2–11.1	1469	155
***LEU2-LYS2*:**									
wild-type	1068	26.6–28.4	496	569	3	4644	25.8–28.4	3388	1256
*csm4Δ*	531	29.7–32.5	216	312	3	2999	28.0–31.3	2110	889
*ndj1Δ*	472	31.3–34.3	192	274	6	2548	28.3–31.9	1782	766
*mlh1Δ*	616	11.8–13.6	459	157	0	3792	11.7–13.8	3309	483
*msh5Δ*	720	11.0–13.0	562	155	3	5674	10.3–11.9	5047	627
*csm4Δ ndj1Δ*	789	31.9–34.9	322	455	12	3836	29.1–32.0	2664	1172
*csm4Δ mlh1Δ*	418	14.4–17.4	295	121	2	4036	14.5–16.7	3407	629
*csm4Δ msh5Δ*	155	10.4–15.4	120	34	1	1624	10.0–13.2	1437	187
***LYS2-ADE2*:**									
wild-type	1068	12.1–13.7	803	263	2	4644	11.8–13.8	4052	592
*csm4Δ*	531	15.3–17.5	362	168	1	2999	16.0–18.7	2480	519
*ndj1Δ*	472	17.8–20.0	294	178	0	2548	16.6–19.7	2087	461
*mlh1Δ*	616	6.2–7.6	531	85	0	3792	6.5–8.1	3517	275
*msh5Δ*	720	3.7–4.7	659	61	0	5674	4.1–5.3	5409	265
*csm4Δ ndj1Δ*	789	18.7–21.3	514	267	8	3836	16.8–19.3	3145	691
*csm4Δ mlh1Δ*	418	6.1–7.7	360	58	0	4036	6.9–8.6	3726	310
*csm4Δ msh5Δ*	155	3.1–5.3	142	13	0	1624	3.1–5.1	1559	65

Relevant genotype	Tetrads^a^					Single spores^b^			
	Number analyzed	cM	PD	TT	NPD	Number analyzed	cM	Parental	Recombinant
***ADE2-HIS3*:**									
wild-type	1068	36.5–38.9	343	709	16	4644	33.3–36.1	3033	1611
*csm4Δ*	531	51.3–57.1	120	378	33	2999	40.2–43.8	1739	1260
*ndj1Δ*	472	53.3–59.9	108	330	34	2548	40.6–44.5	1464	1084
*mlh1Δ*	616	18.2–21.0	400	211	5	3792	16.9–19.4	3104	688
*msh5Δ*	720	17.2–20.2	496	215	9	5674	14.5–16.4	4797	877
*csm4Δ ndj1Δ*	789	52.4–57.4	193	542	54	3836	40.4–43.5	2228	1608
*csm4Δ mlh1Δ*	418	31.5–37.3	215	186	17	4036	23.6–26.3	3031	1005
*csm4Δ msh5Δ*	155	20.0–26.4	93	60	2	1624	14.9–18.6	1354	270
***URA3-LYS2*:**									
wild-type	1068	46.5–49.9	264	759	45	4644	38.0–40.8	2815	1829
*csm4Δ*	531	56.9–63.3	108	380	43	2999	41.7–45.3	1696	1304
*ndj1Δ*	472	59.5–66.9	105	321	46	2548	41.9–45.8	1430	1118
*mlh1Δ*	616	21.8–24.4	351	261	4	3792	20.2–22.9	2976	816
*msh5Δ*	720	15.5–18.1	513	200	7	5674	13.8–15.6	4843	831
*csm4Δ ndj1Δ*	789	56.1–61.1	145	588	56	3836	42.2–45.3	2158	1678
*csm4Δ mlh1Δ*	418	31.7–37.9	222	177	19	4036	23.5–26.2	3033	1003
*csm4Δ msh5Δ*	155	18.7–25.1	97	56	2	1624	16.5–20.3	1326	298
***LYS2-HIS3*:**									
wild-type	1068	46.0–49.6	278	744	46	4644	37.7–40.5	2829	1815
*csm4Δ*	531	64.0–71.2	103	370	58	2999	43.9–47.5	1628	1371
*ndj1Δ*	472	67.5–75.9	100	311	61	2548	43.6–47.5	1389	1159
*mlh1Δ*	616	24.7–28.3	344	261	11	3792	21.7–24.5	2917	875
*msh5Δ*	720	20.5–23.9	465	242	13	5674	17.3–19.3	4638	1036
*csm4Δ ndj1Δ*	789	62.3–68.3	174	532	83	3836	43.0–46.1	2127	1709
*csm4Δ mlh1Δ*	418	33.4–39.0	195	207	16	4036	26.3–29.0	2921	1115
*csm4Δ msh5Δ*	155	24.3–32.5	87	64	4	1624	17.5–21.4	1309	315

All mutants are isogenic derivatives of EAY1108/EAY1112. ^a^Intervals correspond to the genetic distance calculated from tetrads +/- one standard error. Standard error was calculated using the Stahl Laboratory Online Tools website (http://www.molbio.uoregon.edu/fstahl/). ^b^Data shown as 95% confidence intervals around the recombination frequency determined from single spores. To facilitate comparisons to the tetrad data, recombination frequencies obtained from single spore data were multiplied by 100 to yield genetic map distances (cM). The recombination frequency in single spores determined by: Parental/(Parental+Recombinant) and cM indicates the genetic distance in tetrads calculated using the formula of Perkins [38]: 50×{TT+(6×NPD)}/(PD+TT+NPD).

Oh et al. [36] showed that the *sgs1ΔC795* mutation conferred an ∼20% increase of map distance in SK1 strains that was primarily due to an increase in the frequency of NPD tetrads. Based on this observation the authors suggested “… that a fraction of the events that would normally form single crossovers in WT cells gives rise to closely spaced double crossovers in *sgs1ΔC795* cells.” We did not see a similar pattern in *csm4Δ* mutants. Three of four genetic intervals (all but *LEU2-LYS2*) in the SK1 congenic strain displayed significantly different PD:NPD:TT distributions in *csm4Δ* compared to WT, even when the NPD class was ignored (G-test, p<0.05). This observation suggested that the *csm4Δ* mutation did not increase map distances by specifically increasing the frequency of closely spaced double crossovers. This conclusion is reinforced by physical analysis of DNA events: species representing large joint molecules are overrepresented relative to other types of joint molecules in *sgs1Δ* [36] but not in *csm4Δ* (compare Figure 7C with Figure S2C).

### Reduced Crossover Interference in *csm4Δ*

In WT meiosis, occurrence of a CO in one region of a chromosome is accompanied by a reduced probability that one will also occur in a nearby region, a phenomenon known as "CO interference”. We assayed interference in *csm4Δ* by three different methods.

**Figure 4:**
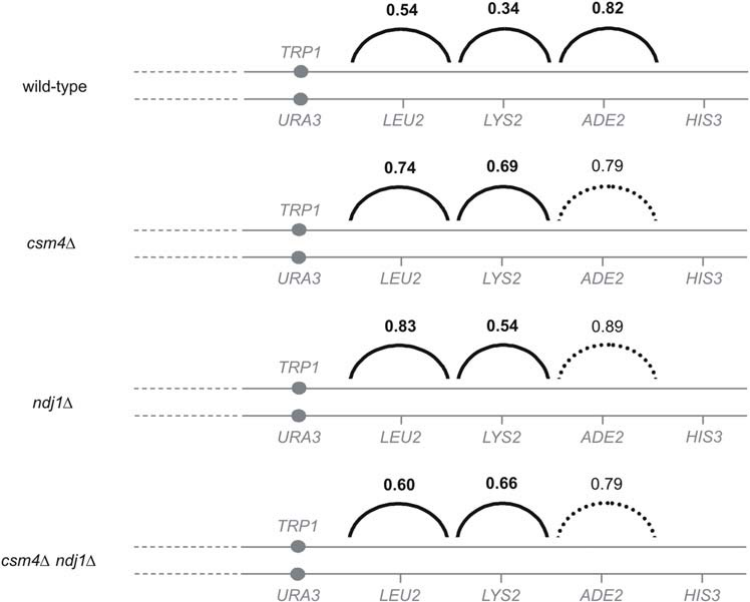
Crossover interference is partially disrupted in *csm4Δ* strains on chromosome XV. Crossover interference was analyzed as described by Malkova et al. [37] and Martini et al. [34] using data obtained from the EAY1108/EAY1112 strain background (Table 1). The numbers above the solid arcs are the average of the significance interference ratios for each interval pair. n.s. and dashed arc indicates that both members of the interval pair did not show significant interference.

One approach utilizes the method of Malkova et al. [37], which evaluates the occurrence of interference in adjacent intervals by utilizing all of the information contained in complete tetrads (Figure 4, Table S3). In WT, interference was observed for all three interval pairs. In contrast, *csm4Δ* strains showed reduced interference in two intervals and no significant interference in the third. One interpretation of these data is that interference does not extend as far from the initial crossover site in *csm4Δ* strains as it does in WT.

A second approach evaluated the “coefficient of coincidence” (COC). For a given pair of intervals, the COC is the ratio of the observed frequency of double CO events to that expected if COs in the two intervals occurred independently. In accord with results obtained using the Malkova et al. [37] method, the *csm4Δ* mutant exhibited a modest reduction in interference in all four intervals analyzed (Table 2).

**Table 2 pgen-1000188-t002:** Interference in WT, *csm4Δ*, *ndj1Δ*, *msh5Δ*, and *mlh1Δ* strains.

Coefficients of coincidence (DCO observed/DCO expected)					Nonparental Ditype Ratios (NPD observed/NPD expected)		
Relevant genotype	*URA3-LEU2-LYS2*		*LEU2-LYS2-ADE2*		*ADE2-HIS3*	*URA3-LYS2*	*LYS2-HIS3*
	Tetrads	Spores	Tetrads	Spores			
wild-type	0.717**	0.799**	0.458**	0.550**	0.215**	0.443**	0.469**
	(177/246.9)	(218/272.9)	(65/141.9)	(88/160.1)	(16/74.5)	(45/101.6)	(46/98.0)
*csm4Δ*	0.905	0.932	0.828	0.910	0.582**	0.676**	0.824
	(176/194.6)	(256/274.8)	(83/100.3)	(140/153.8)	(33/56.7)	(43/63.6)	(58/70.4)
*mlh1Δ*	0.573*	0.649*	0.646	0.628*	0.472	0.250**	0.618
	(19/33.1)	(33/50.8)	(14/21.7)	(22/35.0)	(5/10.6)	(4/16.0)	(11/17.8)
*msh5Δ*	1.184	1.658***	0.747	0.99	0.900	0.833	0.970**
	(20/16.9)	(59/35.6)	(10/13.4)	(29/29.3)	(9/10.0)	(7/8.4)	(13/13.4)
*ndj1Δ*	0.936	0.963	0.729**	0.758**	0.661**	0.820*	0.970
	(166/177.4)	(242/251.3)	(77/105.6)	(105/138.6)	(34/51.4)	(46/56.1)	(61/62.9)
*msh5Δ csm4Δ*	0.791	1.233	0.341	1.069	0.556	0.645	0.870
	(5/6.3)	(22/17.8)	(1/2.9)	(8/7.5)	(2/3.6)	(2/3.1)	(4/4.6)
*mlh1Δ csm4Δ*	1.076	1.175	0.586	0.952	1.056	1.242	0.833*
	(38/35.3)	(108/91.9)	(10/17.1)	(46/48.3)	(17/16.1)	(19/15.3)	(16/19.2)
*ndj1Δ csm4Δ*	0.804**	0.886*	0.793**	0.786**	0.663**	0.577**	0.862
	(215/267.5)	(299/337.3)	(129/162.8)	(166/211.1)	(54/81.4)	(56/97.0)	(83/96.3)

Interference was calculated from data presented in Table 1. The coefficient of coincidence (COC) was examined by a two-tail binomial test from VassarStats (http://faculty.vassar.edu/lowry/VassarStats.html) which tested whether the observed number of double crossovers (DCOs) deviated significantly from the expected number. The expected number of non-parental ditypes (NPDs) and the presence or absence of interference was determined using the two-factor test from the Stahl Laboratory Online Tools website (http://www.molbio.uoregon.edu/fstahl/). Asterisks indicate that interference is present in the interval (^*^ p<0.05; ^**^ p<0.01). ^***^In this interval, although DCOs deviated significantly from the expected number, the COC is greater than 1.

A third approach to interference analysis is the calculation of the ratio of observed non-parental ditypes (NPD) which reflects the occurrence of a four-strand double crossover, to that predicted by the number of single crossovers detected (NPD ratio, [38],[39]). By this criterion, we were unable to determine a difference in interference between WT and *csm4Δ* in all three intervals measured (Table 2). It is not clear why interference as assessed by NPD ratios is less affected by *csm4Δ* than when assessed by other methods. One possible explanation for the disparity between the COC and NPD ratio measurements is that NPD measurements may be affected by “chromatid interference”. Chromatid interference is a restriction on the independence of chromatid selection during CO recombination and has not been previously observed in yeast [37],[40]. Correspondingly, WT and the *csm4Δ* mutant both exhibited a 1∶2∶1 ratio of exchanges involving two, three, or four chromatids in the *URA3-LYS2-HIS3* interval, implying an absence of chromatid interference in both cases (data not shown).

### CSM4 and Crossover-Promoting Factors Act Independently

During meiosis, the formation of COs, as opposed to noncrossovers (NCOs), is promoted by a large number of proteins that are specifically dedicated to this process. Among these, the Msh4-Msh5 complex appears to act around the time of CO/NCO differentiation [41], while Mlh1-Mlh3 appears to act later, likely during double Holliday junction (dHJ) resolution ([42]–[44]; N. Hunter, personal communication). In both the *msh5Δ csm4Δ* and *mlh1Δ csm4Δ* double mutants, recombination levels are significantly lower at all examined intervals than levels seen with the *csm4Δ* alone (G-test, p<0.007, Dunn-Sidak correction, Figure 3, Table 1). This suggests that, while absence of Csm4 affects the level of COs, those COs are still occurring via the normal Msh5/Mlh1-dependent pathway. Conversely, in *msh5Δ* and *mlh1Δ* mutant backgrounds, absence of Csm4 increases CO levels about two-fold above the single *msh5Δ* and *mlh1Δ* mutant levels, suggesting that the effect of *csm4Δ* on CO levels is upstream and/or independent of the *msh5Δ* and *mlh1Δ* effects. Although the *msh5Δ* is only significantly different from its corresponding double mutant at the *URA3-LEU2* interval in the spore dataset, the *mlh1Δ* recombination levels differ significantly from *mlh1Δ csm4Δ* at two out of four intervals in the tetrad dataset and three out of four intervals in the spore dataset (G-test, p<0.025, Dunn-Sidak correction). Furthermore, spore viability in the double mutants (Figure 1; *csm4Δ msh5Δ* = 22%; *mlh1Δ csm4Δ* = 42%) was much lower than any of the single mutants alone (Figure 1; *csm4Δ* = 64%; *msh5Δ* = 36%; *mlh1Δ* = 68%). Taken together, these genetic interactions suggest that Csm4 acts independently of Msh4-Msh5 and Mlh1-Mlh3. Physical analysis of *csm4 msh4* mutants by Kosaka et al. [23] is consistent with this observation.

### Ndj1 and Csm4 Have the Same Function(s) for CO Level and Distribution

*ndj1Δ* conferred a 30–40% increase in CO frequencies at all intervals, indistinguishable from the increase seen in *csm4Δ* (G-test, p<0.007, Dunn-Sidak correction). Crossover interference is also similarly affected in *ndj1Δ* and *csm4Δ*. These unusual phenotypes in both mutants provide strong support for Ndj1 and Csm4 playing similar roles with respect to recombination. In direct confirmation of this conclusion, the *csm4Δ ndj1Δ* double mutant is indistinguishable from either single mutant with respect to increases in CO levels in all four genetic intervals analyzed (G-test, p<0.017, Dunn-Sidak correction, no intervals are significantly different between *csm4Δ* and *csm4Δ ndj1Δ* and only one interval is significantly different between *ndj1Δ* and the double mutant, Figure 3, Table 1) and interference phenotypes (Figure 4; Table 2). We note that a previous study also detected reduced interference in *ndj1Δ* but did not detect increased CO levels [11]. Strain background effects are likely responsible for this difference.

### Absence of Csm4 Does Not Dramatically Increase Non-Mendelian Segregation (Table 3)

Non-Mendelian (non-2:2) segregation of an allele, often referred to as “gene conversion”, implies that a recombination interaction has occurred between homologs rather than sisters. In budding yeast, gene conversion events are usually manifested as 1∶3 or 3∶1 segregation patterns of individual alleles [45]. In the congenic SK1 background, gene conversion levels at *TRP1*, *URA3*, *LEU2*, *LYS2*, *ADE1*, and *HIS3* loci occurred at levels ranging from 0 to 0.8% of tetrads in WT and at indistinguishable levels in *csm4Δ*. The relatively low levels of gene conversion observed for these markers may reflect the fact that they mostly involve 1–3 kb heterologies. We also examined gene conversion at 11 loci marked by a variety of mutation types (point mutations and insertions/deletions). In these SK1 strains [46], non-Mendelian segregation frequencies ranged from 0.2% to 5.3% of tetrads in WT and 0.2% to 5.2% in *csm4Δ* derivatives. The total frequencies of gene conversion at all loci were 14.7% in WT and 17.4% in *csm4Δ*, with no chromosome- or locus-specific differences detectable. Gene conversion frequencies reflect the combined effects of a couple of variables: the frequency of recombination initiation at/near the locus and the probability that an event initiated on one homolog will chose a partner duplex on the other homolog rather than on the sister chromatid. The simplest possibility is that *csm4Δ* has little effect on either of these features, although a balanced effect on both parameters cannot be excluded.

**Table 3:** *csm4Δ* does not significantly affect the percentage of non-mendelian segregation events observed in tetrads.

	Chromosome XV							
	Tetrads	*TRP1*	*URA3*	*LEU2*	*LYS2*	*ADE2*	*HIS3*	All Markers
wild-type	1087	0.0	0.0	0.2	0.6	0.1	0.8	1.7
*csm4Δ*	541	0.0	0.0	0.4	0.6	0.2	0.7	1.8
*ndj1Δ*	482	0.2	0.2	0.4	0.6	0.0	0.8	2.3
*msh5Δ*	757	0.1	0.1	1.6	1.2	0.8	1.2	5.0
*mlh1Δ*	635	0.0	0.2	0.8	0.6	0.5	0.9	3.0
*ndj1Δ csm4Δ*	806	0.0	0.0	0.7	0.4	0.2	0.9	2.2
*msh5Δ csm4Δ*	163	0.0	0.6	2.5	1.2	0.0	0.6	4.9
*mlh1Δ csm4Δ*	452	1.8	1.5	0.9	2.0	1.5	2.0	9.7

	Chromosome III					
	Tetrads	*HIS4*	*LEU2*	*ADE2*	*MATa*	All markers
wild-type	491	2.4	0.4	0.2	0	3.1
*csm4Δ*	559	4.3	0.7	0.2	0	5.2

	Chromosome VII					
	Tetrads	*LYS2*	*MET13*	*CYH2*	*TRP5*	All markers
wild-type	491	1.6	2.9	0.4	0.6	5.5
*csm4Δ*	559	0.9	3.6	0.5	1.4	6.4

	Chromosome VIII					
	Tetrads	*URA3*	*THR1*	*CUP1*		All markers
wild-type	491	0.2	5.3	0.6		6.1
*csm4Δ*	559	0	5.2	0.5		5.7

a Percentage of gene conversion events on chromosome XV in the EAY1108/EAY1112 SK1 congenic background. Of the 167 aberrant segregation events detected, 161 were 3∶1 or 1∶3 single gene conversions and 6 were 4∶0 or 0∶4 double gene conversions. No post-meiotic segregation events were detected.

b Gene conversion percentage on chromosomes III, VII, and VIII in the NH942/NH943 SK1 isogenic background. Of the 169 aberrant segregation events detected, 168 were 3∶1 or 1∶3 single gene conversions and 1 was a 4∶0 or 0∶4 double gene conversion. No post-meiotic segregation events were detected.

### Homolog Nondisjunction in *csm4Δ* Does Not Result from Absence of the Obligatory CO

Homolog disjunction requires the presence of at least one interhomolog connection, created by the combined effects of a CO and the cohesion between sister chromatids centromere-distal to that CO. Homolog disjunction also requires appropriate reductional functioning of homolog centromere/kinetochore complexes and the efficient release of chiasma-maintaining sister connections. Because *csm4Δ* exhibits higher than WT levels of COs, it seems unlikely that homolog nondisjunction in *csm4Δ* results from the absence of a CO. On the other hand, in WT meiosis, special mechanisms ensure that each homolog pair experiences at least one CO (the so-called “obligatory” CO) even when overall CO levels are reduced (for recent discussion see [34]). Thus, it remained possible that Csm4 is required for the occurrence of the obligatory CO.

**Figure 5:**
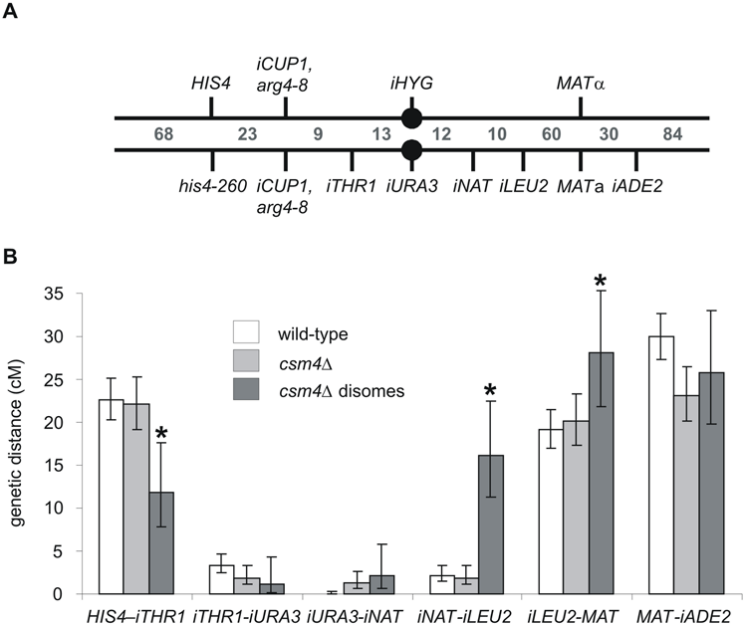
An altered distribution and frequency of crossovers is seen in *csm4Δ* cells that have suffered a chromosome III nondisjunction event. A) Cartoon showing the chromosome III locus in the BR strain background (Materials and Methods). B) Graphical representation of recombination for WT and *csm4Δ* tetrads and *csm4Δ* disomic spores (from data in Table S4). Error bars indicate 95% confidence intervals around the recombination frequency determined from single spores, calculated using VassarStats (http://faculty.vassar.edu/lowry/VassarStats.html). “i” indicates insertion of the indicated marker at an ectopic locus. Recombination frequencies obtained from single spore data were multiplied by 100 to yield genetic map distances (cM). *csm4Δ disomic recombination levels are significantly different (G-test, p <0.025, Dunn-Sidak correction) from the *csm4Δ* tetrad data.

We examined the presence or absence of COs on chromosomes that had undergone nondisjunction using a system developed by Rockmill et al. ([29]; Figure 5, Table S4). This system allows for the selection, purification, and genetic analysis of spores disomic for chromosome III in the BR strain background (Table S1, Materials and Methods). As a baseline for this analysis, map distances for six intervals spanning 167 kb of the 317 kb chromosome III were determined from four-spore viable tetrads in WT (390 tetrads dissected, 317 four-spore-viable, 93% spore viability) and *csm4Δ* (697 tetrads dissected, 203 four-spore viable, 53% spore viability) in the BR strain background. This spore viability pattern observed in *csm4Δ* was similar to that seen for both the congenic and isogenic *csm4Δ* SK1 strains. However, unlike what we observed in the SK1 strain background, the recombination frequencies were similar in WT and *csm4Δ* (only two out of six intervals were significantly different, G-test, p<0.025, Dunn-Sidak correction, see comment on strain background effects below).

Tetrad analysis of the *csm4Δ* derivatives of these strains showed that 9.3% (17/182) of the two-spore-viable tetrads dissected displayed nondisjunction of chromosome III. This value is similar to what was seen in the congenic SK1 strain background (7.8%). Tetrad analysis also revealed that 85% (154/182) of *csm4Δ* two-spore-viable tetrads were sisters, consistent with meiosis I nondisjunction, and again, similar to that seen in the congenic SK1 strain background (88%). These data, along with the spore viability profile (data not shown), show that again, aberrant segregation in *csm4Δ* strains resulted primarily from homolog nondisjunction.

From sporulated *csm4Δ* cultures, we selected and analyzed 185 random spores disomic for chromosome III. In this analysis, CO levels were examined in a manner that accounted for the inability to detect homozygosity of dominant markers in the *csm4Δ* disomic spores and all tetrad information was converted to single spore data to allow direct comparison between the disome and tetrad data (Materials and Methods). Interestingly, the distribution of COs among the examined intervals was significantly different from that observed among chromosomes that experienced regular segregation: when compared to *csm4Δ* tetrads, *csm4Δ* disomes displayed significantly increased levels in the *iNAT-iLEU2* and *iLEU2-MAT* intervals on the right arm of the chromosome and a significantly decreased level of crossing over in the *HIS4-iTHR1* region (G-test, p<0.025, Dunn-Sidak correction, Figure 5, Table S4). In addition, the total map distance for six intervals on chromosome III was higher for the disomes (85 cM) compared to the WT (78 cM) and *csm4Δ* (71 cM) complete tetrads (Figure 5, Table S4). Thus, homolog nondisjunction in *csm4Δ* does not appear to result from absence of the obligatory CO.

More generally, chromosomes are not mis-segregating because of lack of recombination events, too many recombination events, or because they were only receiving crossovers in inappropriate locations (e.g., telomeres, centromeres). The altered distribution of crossovers seen in *csm4Δ* disomes also differed from what was previously seen in disomes isolated from WT and *sgs1Δ* strains. In these backgrounds, elevated levels of crossing over were seen at all loci with the highest levels found at those closest to the centromere, consistent with PSSC causing the majority of the mis-segregation events detected [29]. Rockmill et al. [29] hypothesized that the increase in centromere-proximal crossing over in WT and *sgs1Δ* strains caused PSSC events through the loss of sister chromatid cohesion. However, our data are not consistent with this scenario. The crossovers seen in *csm4Δ* disomes were not consistently higher or lower across the chromosome length, were not localized to a specific chromosomal position (e.g. centromeres), and were clearly not aiding in proper chromosome segregation. Thus there is no clear pattern or trend from these data that can explain how such a changed distribution can cause chromosome mis-segregation. A different type of explanation for homolog nondisjunction is presented below (Discussion).

### Physical Analysis of Recombination

**Figure 6:**
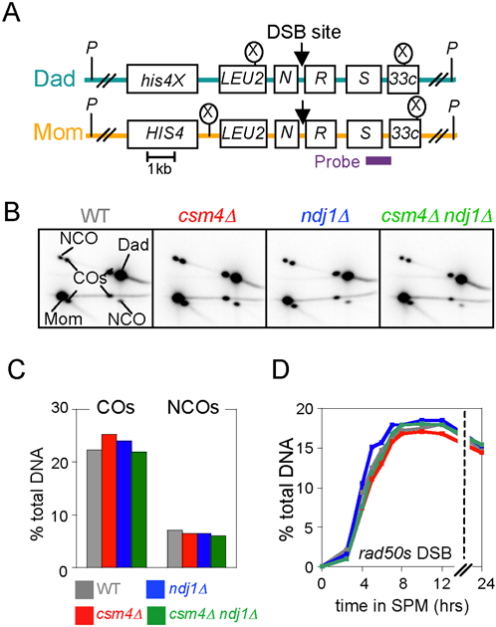
Meiotic recombination analysis at *HIS4LEU2* in WT, *csm4Δ*, *ndj1Δ*, and *csm4Δ ndj1Δ*, Part I. A) Physical map of the *HIS4LEU2* hotspot. Parental homologs, “Dad” and “Mom,” are distinguished via restriction site polymorphisms (circled X = *Xho*I). The locations of the relevant *Xho*I (X), parental, and DSB sites are indicated. B, C) 2D gel analysis of CO and NCO products at *HIS4LEU2.* B 2D gel of CO and NCO tester constructs. *Xho*I digested DNA was electrophoresed in the first dimension gel for 24 h, digested *in situ* with *Bam*HI, and then electrophoresed in the second dimension gel. C) Analysis of CO to NCO products from B. D) Synchronous meiotic cultures of *rad50S-KI81* mutants bearing the *csm4Δ*, *ndj1Δ*, and *csm4Δ ndj1Δ* mutations were analyzed by Southern blot for DSBs at the *HIS4LEU2* locus. Quantitation is shown for gels presented in Figure S3B.

To address the nature of recombination in *csm4Δ/ndj1Δ* meiosis in more detail, we assayed, in synchronously initiated meiotic cultures, physical events at the *HIS4LEU2* locus of chromosome III (Figure 6A), where virtually all events emanate from a single DSB hot spot. Since Csm4 and Ndj1 are implicated in telomere status and dynamics (below), we also asked whether mutant recombination phenotypes depend upon the presence of chromosomal telomeres in *cis* to the assayed locus. For many phenotypes we analyzed recombination between *HIS4LEU2* loci present on circular versions of chromosome III as well as on normal linear chromosomes III. Presence of the circular chromosome was confirmed for all analyzed strains (Figure S3A). All strains examined are isogenic SK1 derivatives (Table S1).

### High Levels of DSBs and CO/NCO Products

**Figure 7:**
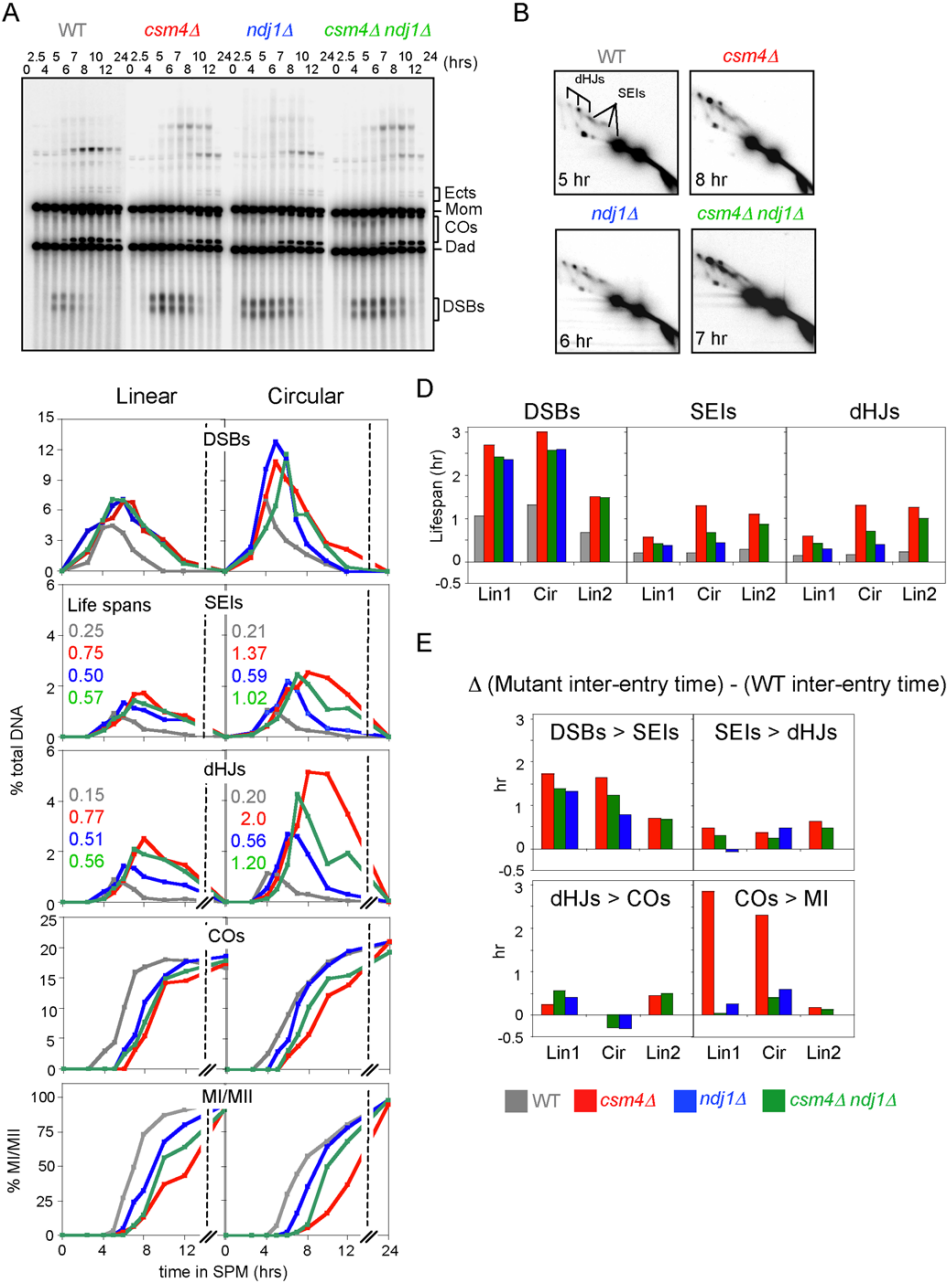
Meiotic recombination analysis at *HIS4LEU2* in WT, *csm4Δ*, *ndj1Δ*, and *csm4Δ ndj1Δ*, Part II. A, B) DNA physical analysis of meiotic recombination in WT, *csm4Δ*, *ndj1Δ*, and *csm4Δ ndj1Δ*. A Synchronous meiotic cultures of the indicated WT and mutant strains analyzed by 1D Southern blot. “Ects” are DNA signal resulting from ectopic recombination involving *HIS4LEU2* and *leu2::hisG* (see [36]). B) 2D gel analysis of recombination intermediates isolated from the indicated WT and mutant strains at times following meiotic induction; same cultures as in A. The positions of single end invasions (SEIs) and double Holliday junctions (dHJs) are indicated by a fork line in the left panel. C) Kinetics of meiotic recombination and MI division in WT, *csm4Δ*, *ndj1Δ*, and *csm4Δ ndj1Δ* containing linear (NKY3890-3893) or circular chromosome III (NKY3894-3897). Levels of DSBs, SEIs, dHJs, and COs are shown for each time point sample as percentages of total DNA. Occurrence of MI shown as percentage of cells that have completed MI (assayed as in Materials and Methods) regardless of whether they have or have not also completed later steps. D) Lifespans of all three assayed intermediates in the two experiments shown in A–C and in a third experiment involving WT, *csm4Δ*, and *csm4Δ ndj1Δ* for the same *HIS4LEU2* alleles (Figure 6A) but a slightly different strain background. E) Analysis of the effects of analyzed mutations on inter-event intervals. For each strain analyzed, a cumulative curve was calculated for entry into the DSB, SEI and dHJ stages, and COs and MI were plotted as % of their maximum levels. From each of these plots, the time at which 50% of cells had “entered the stage” was determined. Then, for each pair of successive events, the interval between the corresponding 50% points was determined. Finally, for each strain examined, and for each such interval, the difference between the interval in the mutant and the interval in WT was determined and plotted.

The levels of CO and noncrossover (NCO) products were determined at the end of meiosis using a two-dimensional gel approach (Figure 6B; [34]). In all three mutants (*ndj1Δ*, *csm4Δ*, and *ndj1Δ csm4Δ*), both types of products were present at high levels (Figure 6C). Correspondingly, DSBs form at the very similar levels in all four strains, as assessed in a *rad50S* background [47] where their turnover to later intermediates is blocked (Figure 6D and S3B). Genetic analysis (above) detected modest increases in COs and non-Mendelian segregations, which presumptively represent total events and thus NCOs as well as COs. Such increases are not obvious in the present study at *HIS4LEU2* (Figure 6C; see also Figure 7C “COs”); however, slightly increased levels of DSBs are reported from analogous analysis of a slightly different version of *HIS4LEU2* by Kosaka et al. [23].

### Delays at Every Post-DSB Step of Recombination

*rad50S* data also show that DSBs occur in a timely fashion in all three mutants, with small differences in timing among different strains that are well within standard culture-to-culture variation (Figure 6D). Since the timing of DSB formation reflects the timing of DNA replication [48] this suggests that DNA replication also occurs with normal timing, which we have confirmed directly in all three mutants by FACS analysis (data not shown).

After DSB formation, however, progression through ensuing steps of recombination is severely delayed, for both linear and circular chromosomes. These steps were analyzed by one-dimensional gels that display DSBs and CO products plus two-dimensional gels that display single-end invasions (SEIs) and double Holliday junctions (dHJs), two branched species on the pathway to formation of CO products ([49],[50],[51]; Figure 7A and B; Figure S3C). All three intermediates (DSBs, SEIs, and dHJs) occur at higher than normal levels and peak at later than normal times in all three mutants, with very similar results for linear and circular chromosomes (Figure 7C). This pattern is indicative of delayed progression, as discussed in detail below. Correspondingly, while CO products form at high levels in all cases, they appear with a substantial delay in all three mutants, for both linear and circular chromosomes (Figure 7C). An additional type of one-dimensional gel analysis of the linear chromosome strains reveals that the same is also true for NCO products, which are delayed to the same extent as CO products in all three mutants (Figure S2A and B).

The detailed effects of *ndj1Δ/csm4Δ* mutations on recombination progression are elucidated by further analysis of the primary data, by two approaches [51]. First, the lifespan of an intermediate, given by the area under the corresponding primary data curve, defines the time spent by a given intermediate at that stage; thus, an increase in the lifespan of a species implies a delay in progression out of the corresponding step. The lifespans of DSBs, dHJs and SEIs all increased in each of the three mutants, relative to WT, for both linear and circular chromosomes, with the biggest increase for DSBs (Figure 7D). Thus, all three mutants confer defects in all three corresponding steps, with the biggest delay in progression out of the DSB stage and lesser delays in progression from SEIs to dHJs and progression from dHJs to COs.

Second, cumulative curve analysis defines the percentage of cells that have “entered” a particular stage as a function of time after initiation of meiosis, with “time of entry” defined as the time at which 50% of cells have carried out the corresponding step. Once again, a delay in progression is seen as an increase in the time interval between the entry time for one step and the entry time for the successive step. Among the three transitions examined, the biggest effect of the mutations is on the difference between the time of DSB formation and the time of SEI formation (i.e. the DSB-to-SEI transition), as expected from lifespan analysis, with smaller (or no) differences seen for the other two other transitions (SEI formation to dHJ formation, dHJ formation to CO formation; Figure 7E). We note that similar effects have been observed not only in the two complete experiments presented in Figure 7C but in a third set of experiments involving a different set of linear chromosome strains (Figure 7D and E, “Lin2”). We also note that while previous work suggested no delay in the DSB-to-SEI transition in *ndj1Δ* [16], reanalysis of that data suggests that the same delay was observed in that study as is reported here.

We conclude that: (i) absence of Ndj1 and/or Csm4 confers delayed progression at every individual assayable step of recombination but most prominently at the DSB to SEI transition; (ii) that linear and circular chromosomes behave quite similarly with respect to these effects, though minor differences are not excluded; and (iii) that delays are not accompanied by any significant reduction (or obvious increase) in the level of final CO and NCO products. There is also a strong tendency for *csm4Δ* to confer the strongest effects among the three analyzed mutations (Figure 7D and E).

Effects of *ndj1/csm4* mutations on three other aspects of recombination were also examined for both linear and circular chromosomes (Figure S2C and D). First, “large joint molecules” (LJMs), indicative of multi-chromatid interactions [36], occur in *ndj1/csm4* mutants as in WT meiosis (Figure S2C). Moreover, direct comparison of LJM and dHJ levels reveals that the two species are affected coordinately, with no indication that the mutants have increased LJM levels as observed in certain other mutants (Figure S2D; [36]). Second, ectopic recombination, which occurs between the molecularly-inserted *LEU2* locus at *HIS4LEU2* and the endogenous *leu2* locus [52], is slightly elevated in all three mutants, as compared to WT, as seen at very late time points (Figure S2C), and as previously observed for *ndj1Δ* [18]. Third, for the linear chromosome, all three mutants exhibit a significant, but somewhat reduced, ratio of inter-homolog versus inter-sister dHJs (∼2.7∶1 versus ∼5∶1 for WT; Figure S2C). This difference could reflect: (i) defective homolog partner choice at the time that choice is made (concomitant with DSB formation; [53], K.K. and N.K. unpublished); (ii) deterioration of homolog bias thereafter; and/or (iii) a differential role of Ndj1/Csm4 in progression of inter-homolog CO interactions versus inter-sister CO interactions. No such difference is observed for the circular chromosome; perhaps this is related to the fact that it does not exhibit such strong inter-homolog bias in WT (Figure S2C).

We note that related analysis of linear chromosome recombination in *csm4Δ* by Kosaka et al. [23] also reveals delays at all assayable steps, very similar to the delays reported here, and, coordinately, delays in formation of COs and NCOs. The two studies differ somewhat with respect to reported effects on the levels of COs and NCOs, perhaps because slightly different assays and *HIS4LEU2* alleles were used. However, in both cases, high levels of both products do occur.

### Recombination-Dependent MI Delay

All three *ndj1/csm4* mutations confer delays in the occurrence of MI. The extent of the delay is greatest for *csm4Δ*, smallest for *ndj1Δ*, and intermediate for the double mutant. This is a highly reproducible effect. It has been observed in both linear and circular chromosome experiments (Figure 7C) and in all of the many other experiments performed with these mutants in the current and previous studies using the SK1 background ([6],[16]; data not shown). In a number of mutants, delayed and/or inefficient occurrence of MI results from delayed recombinational progression. This is also true for *ndj1Δ/csm4Δ* mutants: elimination of recombination initiation completely eliminates the MI delay in all three mutant strains (see below).

Appearance of COs and NCOs marks the end of recombination. Since MI delays are due to delays in recombination, it might be expected that, once these products appear, the mutants should exhibit no further delay in progression. Specifically: occurrence of MI should be delayed to the same extent as occurrence of COs. However, there are hints that this is not the case: occurrence of MI is even further delayed than is occurrence of COs, dramatically for two *csm4Δ* experiments and less dramatically for other mutants and/or other experiments (Figure 7D). Moreover, since all MI delays are completely dependent upon recombination initiation (below), this discrepancy seems to imply that, even after the majority of recombinational interactions are completed (as seen by appearance of the high levels of COs and NCOs as detected by DNA analysis at *HIS4LEU2*), a minority of interactions (which do not make a significant contribution to total DNA-detected events) remain unresolved and are either completed much later or not at all (Discussion).

**Figure 8:**
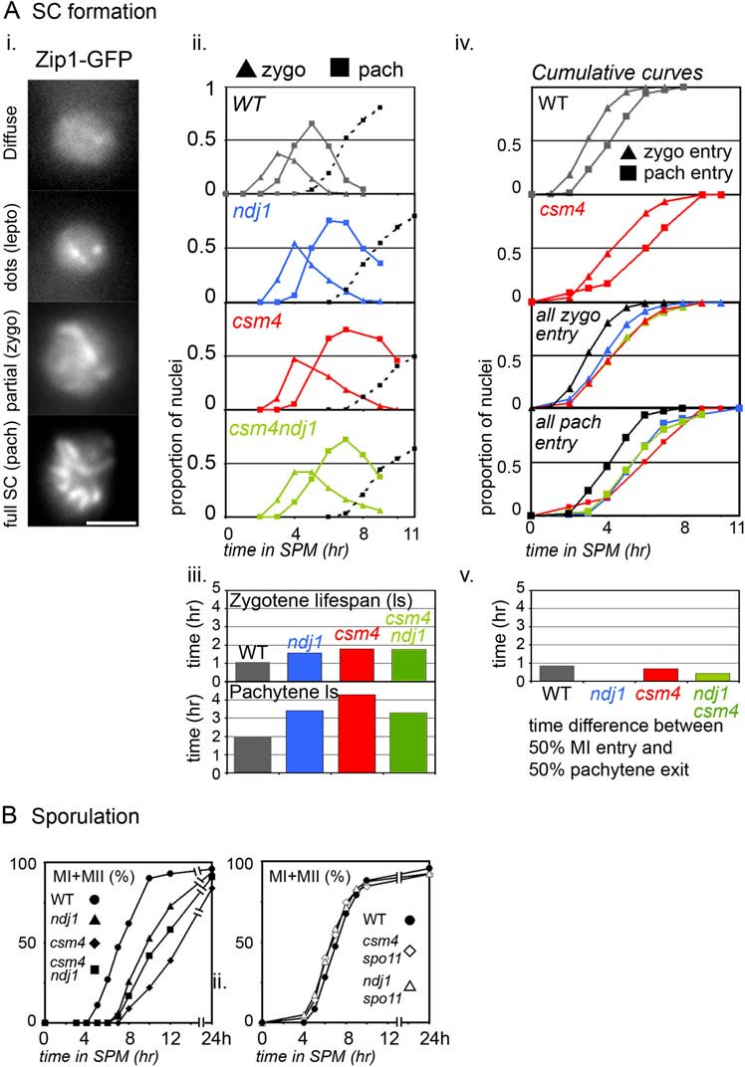
Synaptonemal complex formation and sporulation in *ndj1Δ*/*csm4Δ* mutants. A) Meiotic progression, as measured by the formation of synaptonemal complex (SC). Meiotic progression was monitored in strains where the SC was illuminated by Zip1-GFP(700) (for details, see [2],[6]). i The various steps of SC formation are shown for a WT strain (NKY3834): after diffuse fluorescence, dots appear that correspond to formation of DSBs. Later, short lines correspond to the zygotene onset, whereas at the pachytene stage SC appears as linear, contiguous ribbons. ii For WT, *ndj1Δ*, *ndj1Δ csm4Δ*, and *csm4Δ* strains (NKY3834, NKY3837, NKY4003, and NKY4002, respectively), fixed nuclei where examined at hourly intervals and scored for zygotene (triangle symbol) or pachytene (square symbol) categories. The proportions of nuclei within one or the other categories are plotted as a function of time in SPM. Completion of MI (MI+MII) for each time course is indicated by a dotted line curve. iii zygotene and pachytene lifespan as deduced from the cumulative curve analysis for WT and *ndj1Δ,* and *csm4Δ* mutants. iv Cumulative curve analysis of zygotene and pachytene progression. The upper two panels indicate zygotene and pachytene for WT (top) and *csm4Δ* (bottom). The lower two panels show the onset of zygotene (top) and pachytene (bottom) for all strains. Color code is the same as in ii. v For WT, *ndj1Δ*, *ndj1Δ csm4Δ*, and *csm4Δ* strains, differences between the time when 50% of the nuclei have progressed through MI and the time when 50% have exited pachytene. B) Meiotic progression, as measured by completion of MI divisions in WT (NKY3834), and in *ndj1Δ* and *csm4Δ* mutants without (full symbols, upper panel; strains NKY3837 and NKY4002, respectively) and with the *spo11-Y135F* mutation (empty symbols, lower panel; strains NKY3907 and NKY3908, respectively). All scale bars represent 2 µm.

We further find that the delays in occurrence of MI in all three mutants (Figure 7, also shown in Figure 8B, left panel) are completely eliminated if initiation of recombination is eliminated by the *spo11*(*Y153F*) mutation (Figure 8B, right panel), as seen previously for *ndj1Δ* [16] and for *csm4Δ* by Kosaka et al. [23]. This effect is in accord with the fact that recombination defects trigger MI defects in several other situations (e.g. [41],[54]). For *csm4Δ* we further determined that the MI delay was eliminated by a *rad17Δ* mutation (Figure S4), which is known to alleviate MI delays resulting from recombinational blocks in other situations (e.g. *ndj1Δ*; [55]). Spore viability in *rad17Δ csm4Δ* was dramatically reduced as compared to either single mutant, as would be expected from the compromise of a checkpoint that monitors aberrant recombinational progression [55],[56].

### SC Morphogenesis

Morphogenesis of the SC is readily monitored in whole cells using Zip1-GFP as described previously [41],[57]: cells containing focal Zip1-GFP are in leptotene; those with an incomplete complement of Zip1 linearities are in zygotene, corresponding to formation of SC; and those containing a maximum complement of Zip1 linearities are in pachytene, a morphology corresponding to full length SC (Figure 8A, panel i; e.g. [57]).

*csm4Δ*, *ndj1Δ*, and *ndj1Δ csm4Δ* mutants all exhibit abnormal kinetics of progression into and out of the zygotene and pachytene stages (Figure 8A, panels ii). Lifespan analysis (described above) further shows that all three mutants remain in both stages longer than WT (Figure 8A, panel iii). Cumulative curve analysis (described above) further shows that all three mutants exhibit delayed onset of zygotene and delayed onset of pachytene (Figure 8A, panel iv). These defects can be attributed to defects in progression of recombination (above): onset of zygotene is triggered by CO-designation [58], progression from zygotene to pachytene mirrors the progression of CO-designation and/or SC formation, and exit of pachytene is dependent upon completion of recombination [41],[54]. In accord with these defects, some nuclei (∼20%) exhibit large aggregates of Zip1-GFP, i.e. polycomplexes (data not shown). In our analysis, pachytene appears more prolonged than zygotene; Kosaka et al [23] suggest that zygotene is more severely affected than pachytene. This may represent slight differences in progression in the two experimental protocols or between the particular strains examined.

Noted above, however, was the peculiar fact that, in all three mutants, onset of MI is delayed more than completion of recombination product formation. We favor the idea that there are a small minority of recombinational interactions which persist, undetected by DNA analysis, after most interactions are fully completed (Discussion). If this were true, and given that exit from pachytene is dependent on completion of recombination, and in turn, licenses onset of MI, it could be expected that all mutation-dependent effects would be complete by the end of pachytene, with no further mutant-dependent delay between pachytene exit and MI. This appears to be the case: in all three mutants, exit from pachytene is followed by MI by an interval of time that is the same as, or less than, that observed in WT (Figure 8A, panel v).

## Discussion

The current study: (i) identifies Csm4 as a direct participant in meiotic telomere/NE dynamics with a role that is distinct from that of the previously-identified components of this process; (ii) reveals important roles for Csm4 in both the outcome of recombination, notably in limiting formation of COs and promoting CO interference, and in progression of recombination, notably between DSB formation and onset of stable strand exchange; and (iii) reveals that nondisjunction in the absence of Csm4 is not attributable to absence of COs, perhaps implicating this molecule in the status of intersister connections. The accompanying paper by Kosaka et al. [23] provides related and complementary findings as indicated above.

### Csm4 Is Required for Linkage of Meiotic Telomere/NE Ensembles to the Force Generation System for Chromosome Movement

Our work defines Csm4 as a direct participant in meiotic telomere/NE dynamics, in functional linkage with Ndj1: (i) Csm4 is required for telomere dynamics, similarly to and dependent upon Ndj1-mediated telomere/NE association. (ii) Csm4 partially colocalizes with telomeres along the NE and, correspondingly, deletion of its putative membrane-spanning domain confers a nearly-null phenotype (S. Z. and E. A., unpublished observations). (iii) Similar phenotypes and strong genetic interactions are observed for *csm4Δ* and *ndj1Δ* mutations with respect to recombination, recombination-coupled SC formation, and occurrence of the MI division.

The absence of Csm4 does not discernibly alter meiosis-specific association of telomeres with the NE but strongly abrogates rapid zygotene telomere movements (as well as dynamic telomere-led movements of pachytene chromosomes; [6]), and the tendency for telomeres to colocalize in the vicinity of the SPB at zygotene (the “bouquet”). This latter tendency, seen on a population average basis, likely reflects spatial biasing of rapid telomere movements due to the preferential colocalization of actin cables near the SPB [6]. Moreover, the absence of Csm4 places telomeres in an immobile state that can be partially reinvigorated if meiosis-specific telomere/NE association is absent (with *ndj1Δ*). Telomere-led chromosome movement is dependent upon actin [2],[5],[6]. This movement occurs because of association of telomeres to nucleus-hugging cytoplasmic actin cables which are, themselves, dynamic [6]. Thus, an obvious specific basis for the *csm4Δ* motion defect would be a failure of NE-associated telomeres to become physically and/or functionally coupled to these actin cables.

### Effects of *csm4Δ*/*ndj1Δ* Mutations on Progression of Meiotic Recombination Could Explain the Effects of These Mutations on the Outcome of Recombination

#### CO Patterns Can Be Explained by Increased CO-Designation

Differentiation of recombination intermediates into CO- and NCO-fated types appears to involve a process in which a subset of events is specifically designated for eventual maturation into COs; once this process is complete, remaining interactions are automatically fated for maturation as NCOs as the “default option” [34]. Given this situation, all of the effects of *csm4/ndj1* mutations on CO level and distribution could be explained by an increased number of CO-designation events. (i) The total number of COs (allelic and ectopic) could be increased without a corresponding increase in the total number of recombinational interactions, and without altering the pathway by which CO recombination occurs, as is observed. (ii) As more and more COs occur, the additional events will tend to occur in regions that are less susceptible to CO-designation (and thus still relatively free from the effects of CO interference), thereby altering relative levels along the chromosome. (iii) Continued CO designation would tend to override the inhibitory effects of crossover interference, which would thus appear to be reduced (e.g. [59]). By this scenario, alterations in CO interference, as defined experimentally, would not require any defect in the underlying mechanism by which CO- designation at one site influences the probability of CO-designation at nearby sites. Consistent with this argument, Getz et al. [60] observed a 10–20% increase in crossing over as well as reduced interference at five genetic intervals in *ndj1* mutants (compared to WT, two different strain backgrounds). Based on these and other observations, they conclude that their work offers “evidence of a specific *ndj1*-induced increase in crossovers that are non-interfering... [60].Ó An alternative scenario could involve a primary defect in CO interference which, in turn, would permit additional CO-designation events, e.g. via effects of *ndj1/csm4* mutations on Tel1/Mec1 (ATM/ATR)-mediated signal transduction (e.g. [61]).

#### Increased CO Dsignation Could Be Explained by Prolongation of the CO Designation Period

CO-designation is thought to occur at the end of the DSB stage, after a DSB has found its partner but prior to onset of stable strand exchange, and, thus, at DSB exit [41]. Prolongation of the CO-designation period could explain increased CO levels (and resultant patterning changes) in *csm4/ndj1* mutants. In accord with this model, the DSB stage is greatly prolonged in all three mutants. *csm4/ndj1* SC phenotypes are also in accord with this model. In WT meiosis, each CO-designation event leads to local nucleation of SC installation, which then spreads only a limited distance in either direction (for discussion, see [62]). In *csm4/ndj1* mutants, the period of incomplete SC (zygotene) is prolonged (Figure 8), in accord with occurrence of CO-designation over a longer-than-normal period of time.

#### Prolongation of CO-Designation Could Reflect Defects in Partner Identification or Presynaptic Homolog Juxtaposition

Once a DSB occurs, it must find a homologous partner duplex (on a homolog). The resulting association then mediates the juxtaposition of homolog axes to a close distance of ∼0.4 µm. CO-designation is thought to occur during/after this latter step. In most organisms, these events occur asynchronously throughout the genome of a single nucleus such that partner interaction, homolog juxtaposition, and CO-designation may already have occurred at some loci while, at other loci, DSBs have not yet reached the stage where CO-designation can occur (reviewed in [1],[62]). It would not be surprising if timely exit from the CO-designation period (and thus onset of SEI formation and zygotene) were dependent upon completion of pre-designation events at most or all DSB sites. Correspondingly, prolongation of the CO-designation stage could occur if even a minority of DSBs were significantly delayed in either partner identification or presynaptic homolog juxtaposition.

#### Analogy to the *Drosophila* “Interchromosomal Effect”

The above idea may seem *ad hoc*. However, the alterations in CO patterns observed here for *csm4/ndj1* mutants are strongly reminiscent of the “interchromosomal effect” observed for *Drosophila* [63], a phenomenon in which the presence of a structural heterozygosity (e.g. an inversion on one chromosome relative to its homolog) results in an elevated level of COs plus reduction, but not elimination, of CO interference, i.e. the same phenotype seen in *csm4/ndj1* mutants in the present study. Structural heterozygosity is predicted to delay partner identification and/or immediately ensuing events that require closely proximal coalignment of interacting regions. Thus, this analogy supports the idea that the primary defect in *csm4/ndj1* recombination might occur at these early step(s). Moreover, since in *Drosophila* the irregularity that triggers these effects is confined to the sub-region of the genome affected by the inversion, this analogy supports the notion that, in yeast, late occurrence of immediate post-DSB steps at a minority of DSB sites could trigger a genome-wide effect on CO-designation levels.

#### Delays at Later Stages Could Also Reflect Defects in Early DSB/Partner Interactions

Absence of Csm4/Ndj1 also results in delayed progression of later recombination steps that occur during pachytene, as well as exit from pachytene as observed by SC analysis. These phenotypes could also be explained by a primary defect at early stages by assuming that (i) some uncompleted DSB/partner interactions persist even after most events, and associated SC formation, have been completed and (ii) that these persisting interactions are sensed by the global regulatory mechanisms that permit eventual progression of prophase events through the leptotene/zygotene transition. Support for this idea is provided by the fact that delayed onset of MI in the mutants is dependent upon recombination but tends to be more exaggerated than completion of (bulk) CO formation: such an effect could be explained by “checkpoint” sensing of still incomplete recombinational interactions. Since incomplete recombination results in delayed pachytene exit, which then licenses onset of MI, this interpretation is supported by the fact that no additional mutant-dependent delay is apparent after pachytene.

### *csm4/ndj1* Recombination Defects Could Be a Direct Consequence of Abrogation of Telomere-Led Chromosome Movement

Our work confirms and extends results from analyses of *ndj1Δ* showing that a mutation(s) which affects telomere/NE dynamics also affects meiotic recombination [2],[11],[12],[16]. While it is difficult to be certain that alterations of recombination are a direct consequence of reduced chromosome movement, rather than being a secondary or unrelated effects of altered telomere biology, the current study provides evidence supportive of a direct connection and of a synthetic model for exactly how abrogation of motion might confer such effects.

#### Evidence Pointing to a Cause-and-Effect Relationship

Chromosome movement is strongly reduced in *csm4Δ* and substantially reduced, but to a lesser extent, in *ndj1Δ*. This pattern is readily understood by supposing that the absence of normal meiotic telomere/NE association in *ndj1Δ* releases the chromosomes from more constrained NE/actin cable-associated state found in *csm4Δ*. The same relationship is observed for the delayed occurrence of MI, which in turn is a result of defects in recombination, and is also strongly suggested by DNA analyses of progression at *HIS4LEU2*. Since onset of motion can occur independent of recombination [6], motion would be required for normal progression of recombination rather than the other way around.

#### Motion Could Promote Recombination by Regularizing Topological Relationships among Chromosomes

We have argued elsewhere that the primary role for chromosome movement during meiotic prophase should be the regularization of topological relationships among chromosomes, i.e. removal of chromosomal interlocks or nonspecific connections among unrelated chromosomes [6]. A specific prediction of this hypothesis is that, in the absence of motion, some DSBs within a nucleus may be impeded either from finding a partner region on a homolog or, if a partner is found at the DNA level, from mediating the close juxtaposition of homolog axes to the presynaptic coalignment distance as required for normal recombinosome/axis association and, thereafter, SC formation and continued recombinosome/axis interplay. We have outlined above a scenario in which a failure of a minority of DSBs to identify a partner duplex and/or mediate ensuing homolog juxtaposition could explain the recombination and progression defects of *csm4/ndj1* mutants. Thus, our hypothesis for chromosome movement provides a coherent explanation for the diverse defects of *csm4/ndj1* mutants while, conversely, the phenotypes of *csm4/ndj1* mutants provide circumstantial evidence for our proposal regarding the role of chromosome movement.

#### Explaining Recombination Defects on Circular Chromosomes

The current study presents the intriguing finding that absence of Csm4/Ndj1 affects recombination between circular chromosomes very similarly to recombination between normal linear chromosomes. Formally, this result implies that effects on recombination do not require (or at least are not very strongly dependent upon) the presence of telomeres in *cis* to the affected interacting regions. At first glance, this result would seem to suggest that abrogation of movement is not responsible for recombination defects. However, this is likely not a correct conclusion. Cytological analysis in *S. cerevisiae* of a circular chromosome tagged with a fluorescent repressor/operator array reveals dynamic movement during mid-prophase despite the absence of telomeres (K. Kim, unpublished results). Furthermore, Koszul et al. [6] have found that nearby linear pachytene chromosomes tend to move coordinately despite the absence of telomere clustering at this stage, and the same is presumably true at zygotene. Thus, a defect in the motion of chromosomes possessing telomeres could be transmitted to chromosomes without telomeres, as required by the model proposed above.

#### Other Models

It has often been proposed that telomere movement promotes telomere clustering, which in turn promotes homologous telomere/telomere interactions, which in turn promotes efficient interactions in other regions**.** Homologous telomere/telomere interactions do appear to promote the identification of homologous interactions elsewhere in the genome (e.g. [18],[64]). However, in the case of *S. pombe*, telomere colocalization as such is not sufficient to confer regular recombination; movement is also necessary for some other reason(s) [19].

Another often-proposed model is that motion provides “stirring forces” needed for DSBs to search for and identify homologous partner sequences [65]. Our proposition differs from this idea because it envisions that motion is required (primarily) to eliminate residual topological impediments rather than to positively promote homology searching irrespective of such impediments. One possibility is that homology searching might be promoted by other types of motion which, while less in magnitude, are still significant [6]. Also, once a pair of homologs comes into effective contact at one position (e.g. telomeres; [43]), the problem for further pairing may not be contact between homologous regions as much as making sure that such contacts do not produce entanglements.

There are several arguments against the idea that motion is needed for primary pairing. The most obvious of which is that motion begins concomitant with onset of zygotene [6] which is likely later than the point at which (most) DSBs identify partners. We also note that while mutants with defective telomere/NE ensembles are found to exhibit delayed chromosome “pairing” (e.g. for budding yeast, [11],[12]), the “one spot/two spot” assays used for such studies have significant limitations. First, the level of “one spot” nuclei reflects not only formation of initial contacts but occurrence of events all the way through SC formation, which is certainly delayed in *csm4/ndj1* mutants. Second, given that homologs are periodically connected along their lengths, the level of “one spot” nuclei also reflects chromosome stiffness: greater stiffness results in higher levels of “one spot” nuclei because a contact at one position is propagated farther along the chromosome. And in budding yeast, formation of axial elements, which is likely an indicator of development of chromosome stiffness, normally occurs at the leptotene/zygotene transition [66] and is delayed in *ndj1Δ* [12]. These complexities imply that definitive monitoring of initial DSB/partner interactions requires some different type of assay other than those applied thus far.

### Progression versus Execution

The *csm4/ndj1* recombination phenotypes are different from those conferred by most recombination mutants because they involve delays in progression, at multiple steps, through what appears otherwise to be a normal and efficiently executed process. The existence of this phenotype supports the idea that particular factors are required specifically for timing of events rather than execution. A very similar timing phenotype has recently been described for the budding yeast *pch2Δ* mutant [57], although this mutation confers delays primarily in pachytene events rather than at immediate post-DSB steps. The effects of *pch2Δ* are proposed to be mediated via the regulatory signal transduction kinase Mec1/ATR. The same could be true in the present case, with the addition that earlier events might involve both Mec1/ATR and its relative, Tel1/ATM, which is implicated in events immediately following DSB formation [61].

MI delays of *csm4Δ* (above) and *ndj1Δ* [55] are fully alleviated by elimination of Rad17, implying alleviation of effects triggered by delays at any and all stages of recombination. In accord with action at multiple stages in the current situation, absence of Rad17, or one of its collaborators, is known to alleviate MI arrest conferred by defects at diverse stages of recombination: including DSB exit (*dmc1Δ*; [56]), progression of CO-designated DSBs to later stages (*zip1Δ*; [55],[56]) and timely progression through pachytene (*pch2Δ*; [55]).

Cytological studies suggest that impediments to completion of presynaptic coalignment can also trigger a local response that includes destabilization of chromosome axes around the affected position(s), e.g. at the site of an interlock in *Bombyx* [20] or a structural heterozygosity in mouse [67]. Thus, Rad17-dependent progression delays in *csm4/ndj1* mutants may be part of a standard “checkpoint damage response”. We note, however, that Mec1/ATR and Tel1/ATM are involved in promoting progression of unperturbed WT meiosis, as well as “checkpoint damage sensing” (for discussion see [57]). The same might well be true of Rad17 and its collaborators, in both WT (as shown by Grushcow et al. [52]) and, at least to some extent, in *csm4/ndj1* meiosis. Perhaps these components function to “gate” the signal transduction response such that the rate of progression is appropriately sensitive to the status of the entire population of recombinational interactions in a given nucleus rather than proceeding on a more autonomous clock.

### What Is the Basis for Homolog Nondisjunction in *csm4Δ/ndj1Δ*?

The ultimate raison d'être of meiotic prophase is the proper segregation of homologs at the MI division. This process, in turn, requires the presence of one or more COs between homologous non-sister chromatids. Correspondingly, MI mis-segregation events are often associated with decreased reciprocal recombination levels [25], [68]–[73]). However, the current work provides three lines of evidence that, surprisingly and contrary to earlier presumptions, homolog nondisjunction in *csm4Δ* is not attributable to an absence of COs or, more specifically, to absence of the first “obligatory” CO. First, homologs that have nondisjoined in *csm4Δ* do not exhibit a deficit of COs (Figure 4). A caveat in our analysis is that we were unable to measure telomere distal crossovers in the strains that displayed nondisjunction. Second, the *ndj1Δ* and *csm4Δ* mutations have very similar effects on CO formation while *ndj1Δ* has a much less severe effect on homolog nondisjunction than *csm4Δ*; in the double mutant, it further reduces nondisjunction below the *csm4Δ* level. Third, the primary defect of recombination and downstream events is a temporal delay of a process that eventually proceeded to completion. Nondisjunction events would more likely result from the inefficient execution of a particular process.

One interesting possibility is that some of the COs in *csm4/ndj1* mutants “fail to ensure disjunction” because of a defect in the relationships between sisters. Indeed, there are hints of abnormal sister relationships from detectable increases in PSSC events in these mutants, as shown previously by Conrad et al. [13] in tetrad analysis. Sister relationships are important in three respects: First, sister chromatid cohesion distal to the site of exchange is vital for the stabilization of the physical manifestations of crossing over, chiasmata, which hold the homologous pair together [74]–[76]. Second, at the sites of crossovers, cohesion must be relaxed in order to allow for exchange of the chromosome arms [77]–[79]. Third, sister cohesion along arms distal to the sites of COs must be released during anaphase I. Thus, the *csm4Δ/ndj1Δ* defect could be either a deficit of cohesion or, more intriguingly, a failure of cohesion to be properly released either at the site of the CO, along arms distal to the CO site, or specifically at telomeres.

The scenario presented above, in which a deficit of motion results in defective immediately post-DSB steps of recombination, could also explain a defect in sister relationships. It has recently been shown that CO-designation at leptotene/zygotene is accompanied by local destabilization of chromosome axes; presumably as the first step in differentiation and separation of sister chromatids specifically at these sites [80]. At a site where an initiating DSB fails to establish a normal recombinosome/axis relationship, CO-designation might still occur with respect to DNA events but without accompanying effects on sister relationships. Alternatively, impeded completion of DSB/partner interactions could trigger a local loss of sister connectedness which extends down the chromosome arm(s). Linkage of all *csm4/ndj1* phenotypes to a single common cause is supported by the fact that, for homolog nondisjunction as for other effects, *csm4Δ* confers a stronger defect than *ndj1Δ*.

On the other hand, Csm4/Ndj1 could be involved directly in sister chromatid cohesion, along arms or in centric regions, as an independent aspect of their molecular functions. Indeed, the third component of yeast telomere/NE dynamics, Mps3 has been implicated as a direct general participant in sister chromatid cohesion, in both mitotic and meiotic cells [13],[81].

The *ndj1Δ csm4Δ* double mutant nondisjunction defect is slightly weaker than that of *ndj1Δ*, rather than being the same as or slightly greater than in *ndj1Δ*. Thus, for this phenotype, the defect in each single mutant is subtly dependent upon the presence of the WT gene product corresponding to the other mutation (“partial reciprocal epistasis”). In the context of effects on sister cohesion, a possible explanation is that both mutations have two effects, conferring both a reduction in the number of sister chromatid connections and defective release of those connections that do occur. In this case, each mutation would reduce the number of connections and thus, synergistically, the number of aberrant connections remaining to interfere with MI homolog segregation.

Explanations for homolog nondisjunction that do not involve sister cohesion can also be envisioned. For example, nondisjunction could simply be an additional consequence of the presence of entanglements, which might affect one or more homolog pairs. Alternatively, homolog nondisjunction may result from an excess of COs (e.g. [82]). Our genetic data argue against such a model for *csm4Δ* mutants: we observed that the *csm4Δ* mutation decreased the meiotic viability of *msh5Δ* and *mlh1Δ* mutants (Figure 2) and this would not be expected if it resulted in more COs. A third possibility would attribute homolog nondisjunction to an excess of multi-chromatid events resulting from failure to resolve precursor large joint molecules (e.g. as in *sgs1* [36]). However, there is no evidence that *csm4Δ/ndj1Δ* mutants exhibit a *sgs1*-like defect (above).

### Summary

We construct a coherent model where abrogation of motion confers a defect in completion of early DSB/partner interactions (partner identification or ensuing creation of bridges between homolog axes) which, in turn, explains all other observed mutant defects as described above and in earlier studies. Phenotypes of motion-defective mutants in *S. pombe* have been explained similarly, though in less detail, as a partial defect in recombination and “pairing” [3],[9],[19]. However, diverse alternative explanations for some or all of the observed effects are not critically excluded. Future studies must now critically address predictions of this model, for yeast and for other organisms, e.g. assessment of local DSB/partner interactions and occurrence of aberrant topological relationships, on a per-cell basis.

## Materials and Methods

### Media, Strains, and Plasmids

Yeast strains are listed in Table S1. Strains were grown in either yeast extract-peptone-dextrose (YPD) or minimal selective media [83]. Sporulation plates were prepared as described previously [84]. All incubations were performed at 30°c for the experiments presented in Figures 1, 3, 4, 5, and S4, Tables 1–3, and Tables S1, S2, S3 and S4. When required, geneticin (Invitrogen), nourseothricin (Hans-Knoll Institute fur Naturstoff-Forschung), and hygromycin B (Calbiochem) were included in YPD media as described [85],[86]. Plasmids and integrating vectors were introduced into yeast strains using standard methods [87].

The EAY1108 and EAY1112 SK1-congenic strains were described in Argueso et al. [27] and the NH942 and NH943 SK1-isogenic strains were described in de los Santos et al. [46]. The BR4635-8Bα and BR4256-5Ba strains are derivatives of those described in Rockmill et al. [29]. This BR strain set was used because it was specifically designed to measure crossing over on a chromosome that had experienced a nondisjunction event [29]. All diploids homozygous for coding region deletion mutations in *CSM4*, *NDJ1*, *MSH5*, *MLH1*, and *RAD17* were created by sequential transformation of the parental strains and the mutations were marked with the *KANMX4*, *NATMX4*, or *HPHMX4* as shown in Table S1 [85],[86]. Details on how the mutations were introduced into these strains are available upon request.

*CSM4* was mutagenized by overlap PCR [88] to create the single-step integrating plasmid bearing the N-terminal GFP-Csm4 integrating vector pEAI242. Details on how this plasmid was made are available upon request. pEAI242 was linearized with *Sac*I and *Sph*I prior to transformation. We tested the functionality of the N-terminal GFP-Csm4 construct by integrating it into the EAY1108/EAY1112 background where WT displays 97% spore viability (n = 1199 tetrads) and *csm4Δ* displays 64% spore viability (n = 1164). The integration strains displayed 92% spore viability (n = 40), indicating that the GFP-Csm4 fusion is functional.

#### Tetrad Analysis

Diploids derived from EAY1108/EAY1112 and NH942/NH943 were sporulated using the zero growth mating protocol [89]. Briefly, haploid parental strains were patched together, allowed to mate for 4 h on complete minimal plates, and then transferred to sporulation plates where they were incubated at 30°c for 3 days. Tetrads were dissected on minimal complete plates and then incubated at 30°c for 3–4 days. Spore clones were replica-plated onto relevant selective plates and assessed for growth after an overnight incubation. EAY1871-1873 diploids derived from BR4635-8Bα/BR4256-5Ba were created by mating *MATa* and *MATα* haploids overnight on YPD and then identifying zygotes.

Genetic map distances were determined by the formula of Perkins [38] and the expected number of non-parental ditype tetrads (NPD) was calculated using the equation of Papazian [39]. Interference calculations from three-point intervals were conducted as described [46], [90]–[92]. Statistical analysis was done using the Stahl Laboratory Online Tools (http://groik.com/stahl/) and VassarStats (http://faculty.vassar.edu/lowry/VassarStats.html) and the Handbook of Biological Statistics (http://udel.edu/mcdonald/statintro.html). Interference was measured by the Malkova method [34],[37]. When multiple statistical comparisons using the same dataset were made, we applied the Dunn-Sidak correction as described in Martini et al. [34] and Hoffman et al. [35]. For example, three comparisons were made using the *ndj1Δ* data from the congenic strain background (*ndj1Δ* versus WT, *csm4Δ*, and *ndj1Δ csm4Δ*) therefore p values must be below 0.017 to be considered significant. Comparisons of map distances between disomes and tetrads were done by converting data from complete (non-aberrant) tetrads into single spore data. The WT, *mlh1Δ*, and *msh5Δ* data presented in this paper were published previously in Argueso et al. [27].

#### Disome Selection Assay

Hyg^R^ Ura^+^ spores were selected from purified spores obtained by sporulating EAY1873 (WT) and EAY1871 (*csm4Δ*) as described in Rockmill et al. [93]. Hyg^R^ Ura^+^ diploids that escaped this selection were subsequently eliminated from further study because they could be induced to enter meiosis and form spores that fluoresced when exposed to UV light (254 nM [94]). In contrast, haploid spores disomic for chromosome III can enter meiosis, but do not form spores. In this assay, the Hyg^R^ Ura^+^ clones obtained from the spore purification procedure were replica plated from vegetative media onto a sporulation plate overlayed with a nitrocellulose filter. Cells were sporulated for 3–4 days at 30°c, and then screened using UV light to eliminate diploids as described above. Remaining Hyg^R^ Ura^+^ clones that also tested positive for disomy based on an Arg^+^ phenotype were scored for crossover events as described [29]. Briefly, crossovers in specific intervals were detected based on the following criteria: 1. *HIS4-iTHR1* interval-disomic spore clones required histidine but not threonine for growth, or vice-versa. 2. *iTHR1-iURA3* interval-disomic spore clones required threonine for growth. 3. *iURA3-iNAT* interval-disomic spore clones were sensitive to nourseothricin. 4. *iNAT-iLEU2* interval-disomic spore clones required leucine for growth but were resistant to nourseothricin, or vice-versa. 5. *iLEU2-MAT*-disomic spore clones did not require leucine for growth and were able to mate, or vice-versa. 6. *MAT-iADE2*-disomic spore clones that were unable to mate and required adenine, or vice-versa. Recombination values were multiplied by two to account for the inability to detect disomes homozygous for dominant markers. Recombination frequencies obtained from single spore and disome data were multiplied by 100 to yield genetic map distances (cM). In these strains, “i” refers to insertion of the indicated marker at an ectopic location. Disomes were only compared to complete tetrads because we were interested in comparing spores that had undergone a mis-segregation event to those that had not.

#### Meiotic Time Courses and Physical Assays

Yeast pregrowth and synchronous sporulation were performed as described [51] except that all media were preequilibrated at 30°c prior to use. The synchrony of meiosis was monitored by measuring pre-meiotic DNA replication (FACS analysis) and the progression of MI and MII divisions (DAPI staining [66],[95]). Physical analysis of chromosomal DNA isolated in the meiotic time courses presented in Figures 6, 7, S2 and S3 was performed as described [34],[41],[51]. DNA species identified in one-dimensional (1D) and two-dimensional (2D) gel electrophoresis were quantified using a Bio-Rad phosphoimager and QuantityOne software. The timing of DSB, SEI and dHJ intermediates was evaluated using a life span program kindly provided by Neil Hunter. Analysis of linear and circular versions of chromosomes III by pulse-field gel electrophoresis (PFGE) was performed according to the Bio-Rad CHEF instruction manual (1% Seakem Gold agarose gel at 14°c, 6V/cm, switch times of 60- to 120-sec, and a switch angle of 120 degrees). For every parameter analyzed, data are presented for cultures that have carried out WT or mutant meiosis with “characteristic kinetics” as defined by FACS, Zip1-GFP or MI division analysis of many cultures over time (hundreds for WT and tens for each mutant). Experience tells us that careful analysis of one really good time course is worth many repetitions of less good time courses and that the variability from day-to-day is no different from the variability from culture-to-culture on the same day. Thus, detailed analysis of suitable single cultures is thus presented. However, every finding emphasized above as a significant result emerging from time course analysis has been observed in two or more independent experiments.

#### CSM4 Immunofluorescence

Cell samples were taken at hourly intervals over meiotic time courses of strains EAY1797 and NKY4005. Cells were fixed in formaldehyde and prepared for immunofluorescence using previously described methods [96]. Rap1-RFP was imaged using a Texas Red filter set. Csm4-GFP was visualized using rabbit anti-GFP antibodies (Invitrogen) followed by goat anti-rabbit Alexa488 (Invitrogen). 20 Z-sections of 0.2 µm were taken of each field of cells for RFP, Alexa488 and DAPI. Appropriate z-sections were compared to assess localization of the proteins.

#### Cell Imaging

Cells were observed at room temperature using an epifluorescence microscope (Zeiss) equipped with GFP, DAPI and TexRed filters, a Cascade 512b CCD camera (Roper Scientific), and a PIFOC piezo device (Physik Instrumente) to drive a 100X oil immersion objective (NA 1.45) for acquiring Z-stacks. Images were acquired using Metamorph software.

*Live-cell:* Cells samples of *RAP1-GFP* meiotic cultures were vortexed at full speed for 10 sec and 3–4 µl of suspended cells were rapidly spread onto a glass slide (plain, non-treated) as described [6]. Briefly, for telomere disposition analysis, Z-stack time-series were recorded at a distance of 0.4 µm between each plane (10 planes total, 1.2 sec intervals, 900 ms exposure), every 15 sec for 1 min. For telomere 2D tracking, the focal plane was positioned at the top of each nucleus, and the Rap1-GFP signal was acquired at one-second intervals over 1 minute (exposure time 700 ms).

*Fixed-cell:* Rap1-GFP, Spc42-yECFP (except *ndj1Δ* NKY3906 cells containing only Rap1-GFP) cell aliquots were sampled at hourly intervals after transfer to sporulation media and crosslinked with 1% formaldehyde for 1 hr on ice. Tris HCl pH 7.4 was added to 50 mM final concentration. Samples were incubated on ice for one hour, and then centrifuged in a microcentrifuge for 5 sec at full speed, resuspended in water and stored at 4°c overnight. Cells were then spread onto glass slides and series of z-stack pictures, ∼100–150 cells per time point, were analyzed (0.2 µm×15 frames with 900 ms exposure for the GFP signal, and 0.4×10 frames with 900 ms exposure for the RFP signal).

### Image Analysis

All images were analyzed using ImageJ [97] and/or Metamorph functions. Deconvolution of 2D and 3D acquisitions was performed using AutoDeblur.

#### Tracking of LacO-Telomeres in Live-Cells

To overcome technical difficulties incurred by 3D time-lapse recording of dynamic telomeres, Rap1-GFP spots present in the focal plane of nuclei tops were tracked over time until they moved out of focus. The X- and Y-coordinates of the LacI-GFP spot centroid were determined using the SpotTracker2D ImageJ Plug-in [32] when movement was limited, and manually in WT (t = 4 h). Spot relocation between two successive frames was calculated and apparent velocity was deduced. The apparent velocity of a spot observed in this single focal plane was assumed to be a reasonable approximation of actual velocity. 5 to 12 foci from at least 5 independent nuclei were tracked for 10 to 60 sec, yielding to a minimum of 340 one-second step-sizes for WT, 4 h and up to a maximum of 1200 measurements for *ndj1Δ csm4Δ*, 2 h (most other sets of data comprise between 580–800 measurements). For statistical convenience, the step-size histograms of x, y coordinates displacement of Rap1-GFP spots were constructed. All the data sets, except the one corresponding to WT t = 4 h, exhibit a distribution close to a Normal distribution, as revealed by the use of a Kolmogorov-Smirnov goodness-of-fit test (http://www.physics.csbsju.edu/stats/KS-test.html, 1% level; for each data set a relatively small (∼5–10%) subset of points diverge from the hypothesized continuous distributions, as compare to WT t = 4 h (∼35%). In order to perform further parametric tests between data sets, and because of the closeness to Normal distribution the assumption of Normality was postulated for all distributions, but WT at t = 4 h. Mean velocities and variances were compared using t-test and f-test, respectively (significance level 5%).

#### Telomere Localization in Live Cells

3D time-lapses of prophase cells (0.4 µm×10, every 15 sec for 1 min) were deconvoluted and the position of telomere foci in each plane was visually monitored (comparison of focal planes over time improve the detection of telomere foci) and nuclei where categorized either in the “peripheral” or in the “dispersed” class, depending on the absence of presence of spots within the nuclear volume, respectively (Figure 2B, panel i). The position of the nuclear periphery (corresponding approximately to the position of the nuclear membrane) was defined as the limit of the diffuse GFP signal that result from global Rap1-GFP proteins binding to chromatin.

#### Bouquet Formation in Fixed Cells

Telomere organization was examined in formaldehyde-fixed nuclei by manual inspection of complete series of 3D sections (top to bottom of 15×0.2 µm z-series). All nuclei contain many bright Rap1-GFP foci representing single and/or coalesced telomeres. Nuclei were first scored with respect to whether bright Rap1-GFP foci did or did not exhibit full peripheral localization (defined in Figure 2A). Nuclei without peripheral localization are, by definition, not in the bouquet stage. For nuclei in which all bright Rap1-GFP foci are NE-associated, inspection of the entire set of images makes it possible, with only rare exceptions, to reproducibly assign each nucleus to a condition in which those foci either tend to occur in a single sub-region of the NE (Figure S1A, not quantitatively but qualitatively approximated to be ∼1/4 of the surface area), or give no evidence of such colocalization (Figure S1B). In the former case, there is a further distinction as to whether the colocalization region is, or is not, near the SPB. For this analysis we have defined the bouquet “rigorously” as colocalization in the vicinity of the SPB (illuminated by Spc42-RFP; inspection from top to bottom of 10×0.5 µm z-series), although there is reason to suspect that this is not an absolute requirement for defining this stage [6]. Among bouquet nuclei, an additional distinction can be made as to whether virtually all signals colocalize in a single region (“tight bouquet”, Figure S1, panel Ai, SPB position indicated by a turquoise line on the 2D projection image above) or whether a significant fraction of signals are present outside the main area of colocalization (“loose bouquet”, Figure S1, panel Aii). This same categorization has been made in other organisms (e.g. *Sordaria*, D. Zickler, personal communication) and in our earlier work (O. Nanassy and N.K., unpublished). For the *ndj1Δ* mutant (NKY3906), the SPB is not labeled, however almost no “bouquet” clusters were observed as any time. 100–150 nuclei were analyzed at each time point.

#### Movements of Zip1-GFP Illuminated Chromosome

Time-lapse series of pachytene nuclei from strains expressing Zip1-GFP(700) were recorded at one second intervals over one minute. All the nuclei movies of all the strains, including WT, were pooled and assigned random names. They were subsequently categorized by “blind testers” within three groups according to the amount of observable chromosomal motion (fast motion corresponding to pachytene WT nuclei, the other two categories reflecting two type of nuclei exhibiting hardly or little motion, respectively). Results clearly revealed a difference between *csm4Δ* and *ndj1Δ* mutants. Double mutant was more ambiguous, and will necessitate quantitative analysis to be interpreted.

## Supporting Information

Figure S1 Bouquet classification. Same nucleus as in Figure 2B, panel i, of cells expressing Rap1-GFP and categorized according to whether or not those foci either (A) tend to occur in a single sub-region of the NE or (B) give no evidence of such colocalization. For each nucleus, the 2D projections of the complete series of 3D sections of nuclei showing either Rap1-GFP (15 frames, bottom left) or Spc42-RFP signal (10 frames, bottom right). In the case of A, there is a further distinction as to whether the colocalization region is, or is not, near the SPB (indicated with the turquoise line in the 2D projection) allowing further categorization in “tight bouquet” (i) or “loose bouquet” (ii). All scale bars represent 2 µm.
(3.8 MB TIF)

Figure S2 Further analysis of recombination in WT, *csm4Δ*, *ndj1Δ*, and *csm4Δ ndj1Δ* strains. A, B) Formation of COs and NCOs were assayed by the approach of Storlazzi et al. [98]. This method monitors the appearance of two species which, in WT meiosis, are known from tetrad analysis to arise specifically in association with CO and NCO recombination (“COs” and “NCOs”; Panel A, top). Appearance of both types of products is delayed in *csm4/ndj1* mutants (Panel A, bottom) in accord with appearance of COs as observed by standard one-dimensional gel analysis (Figure 7 legend). When the levels of the two types of products are compared directly, by plotting levels as “percentage of the maximum level”, it is further seen that the two types of products are delayed almost identically (Panel B). It can also be noted that the levels of both the CO and NCO species are reduced in the mutants as compared to WT (Panel A, bottom). The basis for this effect, which is not seen by other types of product analysis (Figures 6 and 7 legends) is unknown. However, detection of products in this assay is specifically dependent upon the way that heteroduplex DNA at the DSB site is formed and its mismatches repaired [98]. Thus, it could be the case that *ndj1/csm4* mutants affect one or both of these processes. C) Quantification of large joint molecules (LJMs) from 2D gels, and ectopic recombination from 1D gels, and the ratio of interhomolog dHJs to intersister dHJs as determined from 2D gels. D Direct comparison of LJMs and dHJs with normalization to maximum level of LJMs in *csm4Δ*, showing that the two species are affected identically in all mutant situations.
(0.8 MB TIF)

Figure S3 DSB formation and meiotic recombination analysis of *HIS4LEU2* hotspot in WT, *csm4Δ*, *ndj1Δ*, and *csm4Δ ndj1Δ* strains. A) Pulse-field electrophoresis gel showing the migration of linear and circular chromosome III in linear and circular chromosome III strains of each genotype, respectively (Table S1). B) Synchronous meiotic cultures of *rad50S-KI81* mutants bearing the *csm4Δ*, *ndj1Δ*, and *csm4Δ ndj1Δ* mutations (Table S1) were analyzed by Southern blot for DSBs at the *HIS4LEU2* locus. The probe shown in Figure 6 was used for hybridization. C) Synchronous meiotic cultures of WT, *csm4Δ*, *ndj1Δ*, and *ndj1Δ csm4Δ* strains bearing a circular chromosome III examined by Southern blot for recombination species present at the *HIS4LEU2* locus. DSBs, COs and ectopic recombination products (Ects) were quantified from 1D gels; SEIs, IS-dHJs, and IH-dHJs were quantified from 2D gels. The hybridization probes and Southern blot methodologies were the same as described in Figure 6. †, meiosis-specific cross hybridizing signal.
(2.3 MB TIF)

Figure S4 *csm4Δ* confers a defect in meiotic progression that is suppressed by the *rad17Δ* mutation. Synchronized meiotic cultures of WT (diamond, EAY1553), *csm4Δ* (square, EAY1554), *rad17Δ* (triangle, EAY2201) and *csm4Δ rad17Δ* (cross, EAY2202) were analyzed for the completion of at least MI (MI+MII) as measured by DAPI staining. A representative experiment is shown. Tetrads dissected from sporulated strains displayed the following percent spore viability: WT-93% (Figure 1), *csm4Δ*-65% (Figure 1), *rad17Δ*-17% (175 tetrads dissected), and *csm4Δ rad17Δ*-1.1% (87 tetrads dissected).
(0.07 MB TIF)

Table S1 Strains used in this study.
(0.1 MB DOC)

Table S2 Genetic map distances (cM) and the distribution of parental and recombinant progeny for the NH942/NH943 strain background in WT and *csm4Δ* on chromosomes III, VI, and, VIII.
(0.1 MB DOC)

Table S3 Interference as measured by the Malkova method.
(0.2 MB DOC)

Table S4 Crossing over in WT tetrads, *csm4Δ* tetrads, and *csm4Δ* disomic spores.
(0.03 MB DOC)
