# Supplementary figures and images for: Csm4, in Collaboration with Ndj1, Mediates Telomere-Led Chromosome Dynamics and Recombination during Yeast Meiosis
Source: PLoS Genet. 2008 Sep 26;4(9):e1000188. doi: 10.1371/journal.pgen.1000188 (PMC2533701; doi:10.1371/journal.pgen.1000188)

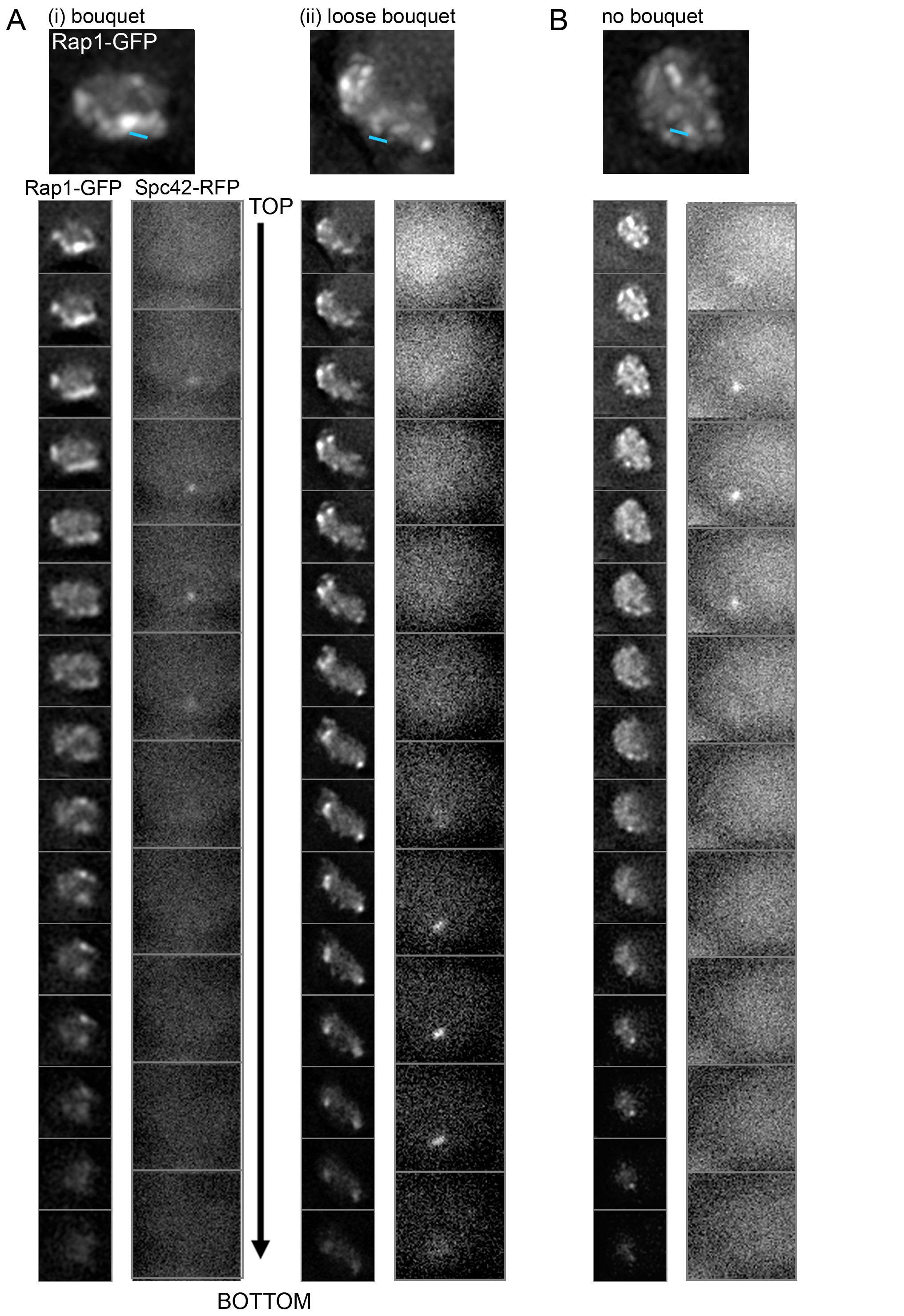

Supplement: Figure S1 — Bouquet classification. Same nucleus as in Figure 2B, panel i, of cells expressing Rap1-GFP and categorized according to whether or not those foci either (A) tend to occur in a single sub-region of the NE or (B) give no evidence of such colocalization. For each nucleus, the 2D projections of the complete series of 3D sections of nuclei showing either Rap1-GFP (15 frames, bottom left) or Spc42-RFP signal (10 frames, bottom right). In the case of A, there is a further distinction as to whether the colocalization region is, or is not, near the SPB (indicated with the turquoise line in the 2D projection) allowing further categorization in “tight bouquet” (i) or “loose bouquet” (ii). All scale bars represent 2 µm. (3.8 MB TIF) [file pgen.1000188.s001.tif]

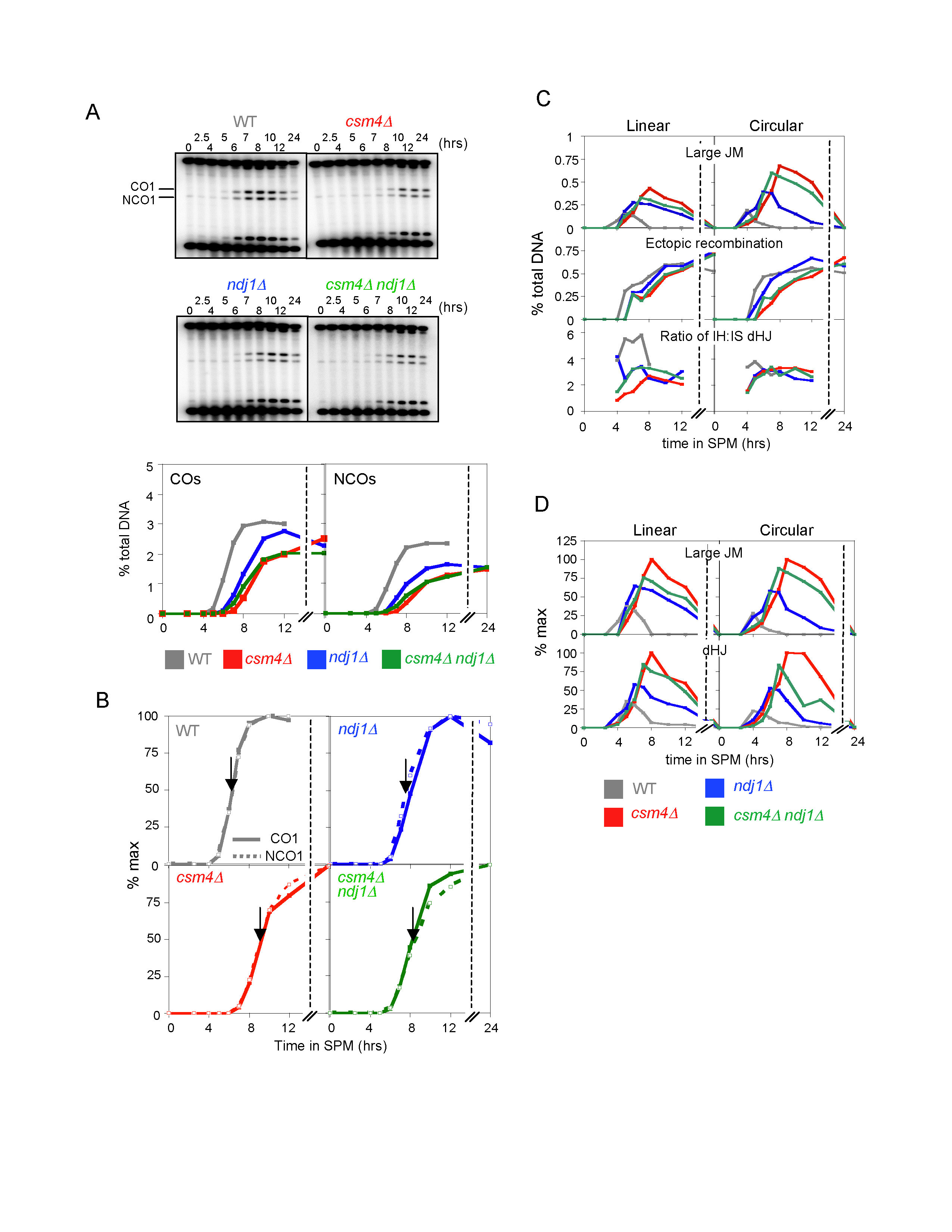

Supplement: Figure S2 — Further analysis of recombination in WT, csm4Δ, ndj1Δ, and csm4Δ ndj1Δ strains. A, B) Formation of COs and NCOs were assayed by the approach of Storlazzi et al. [98]. This method monitors the appearance of two species which, in WT meiosis, are known from tetrad analysis to arise specifically in association with CO and NCO recombination (“COs” and “NCOs”; Panel A, top). Appearance of both types of products is delayed in csm4/ndj1 mutants (Panel A, bottom) in accord with appearance of COs as observed by standard one-dimensional gel analysis (Figure 7 legend). When the levels of the two types of products are compared directly, by plotting levels as “percentage of the maximum level”, it is further seen that the two types of products are delayed almost identically (Panel B). It can also be noted that the levels of both the CO and NCO species are reduced in the mutants as compared to WT (Panel A, bottom). The basis for this effect, which is not seen by other types of product analysis (Figures 6 and 7 legends) is unknown. However, detection of products in this assay is specifically dependent upon the way that heteroduplex DNA at the DSB site is formed and its mismatches repaired [98]. Thus, it could be the case that ndj1/csm4 mutants affect one or both of these processes. C) Quantification of large joint molecules (LJMs) from 2D gels, and ectopic recombination from 1D gels, and the ratio of interhomolog dHJs to intersister dHJs as determined from 2D gels. D Direct comparison of LJMs and dHJs with normalization to maximum level of LJMs in csm4Δ, showing that the two species are affected identically in all mutant situations. (0.8 MB TIF) [file pgen.1000188.s002.tif]

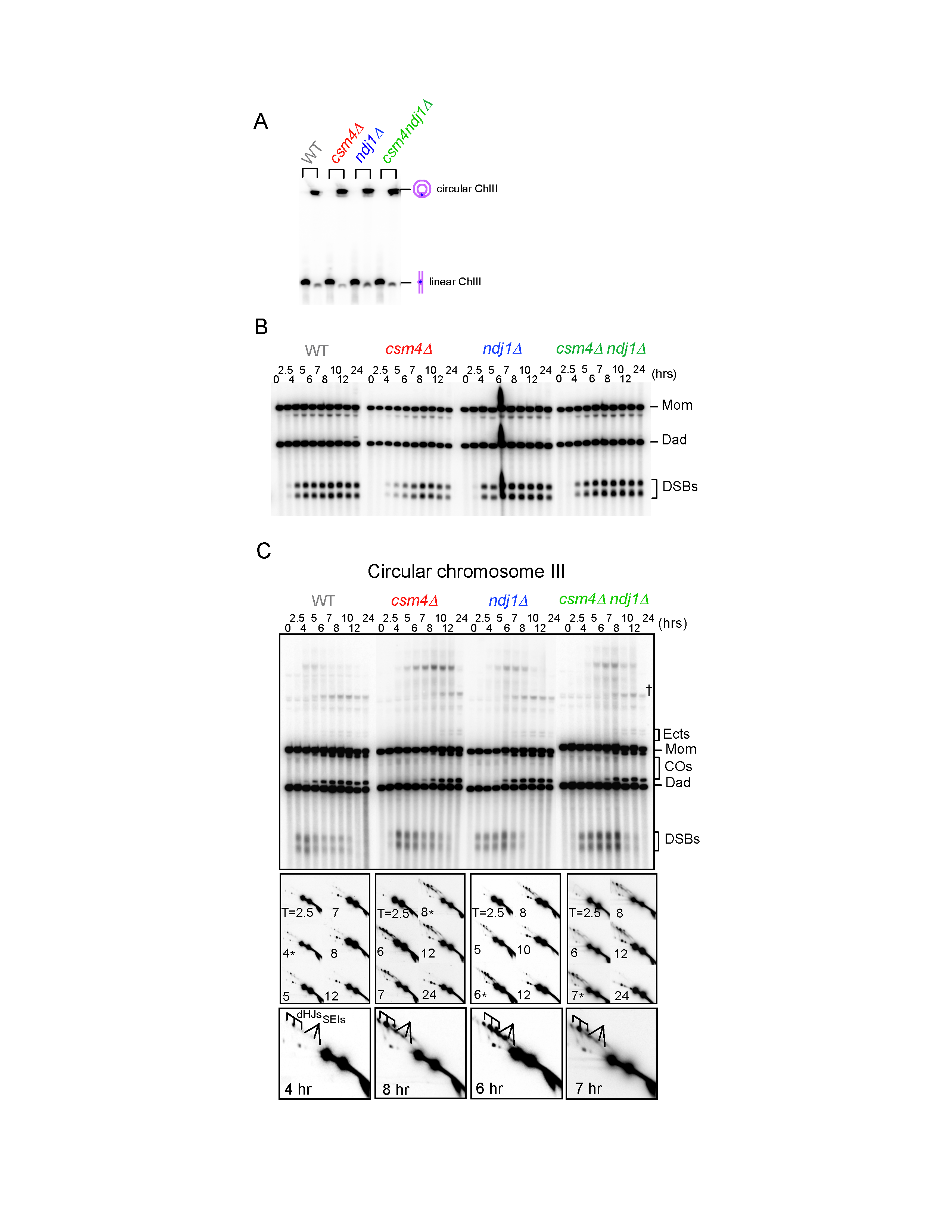

Supplement: Figure S3 — DSB formation and meiotic recombination analysis of HIS4LEU2 hotspot in WT, csm4Δ, ndj1Δ, and csm4Δ ndj1Δ strains. A) Pulse-field electrophoresis gel showing the migration of linear and circular chromosome III in linear and circular chromosome III strains of each genotype, respectively (Table S1). B) Synchronous meiotic cultures of rad50S-KI81 mutants bearing the csm4Δ, ndj1Δ, and csm4Δ ndj1Δ mutations (Table S1) were analyzed by Southern blot for DSBs at the HIS4LEU2 locus. The probe shown in Figure 6 was used for hybridization. C) Synchronous meiotic cultures of WT, csm4Δ, ndj1Δ, and ndj1Δ csm4Δ strains bearing a circular chromosome III examined by Southern blot for recombination species present at the HIS4LEU2 locus. DSBs, COs and ectopic recombination products (Ects) were quantified from 1D gels; SEIs, IS-dHJs, and IH-dHJs were quantified from 2D gels. The hybridization probes and Southern blot methodologies were the same as described in Figure 6. †, meiosis-specific cross hybridizing signal. (2.3 MB TIF) [file pgen.1000188.s003.tif]

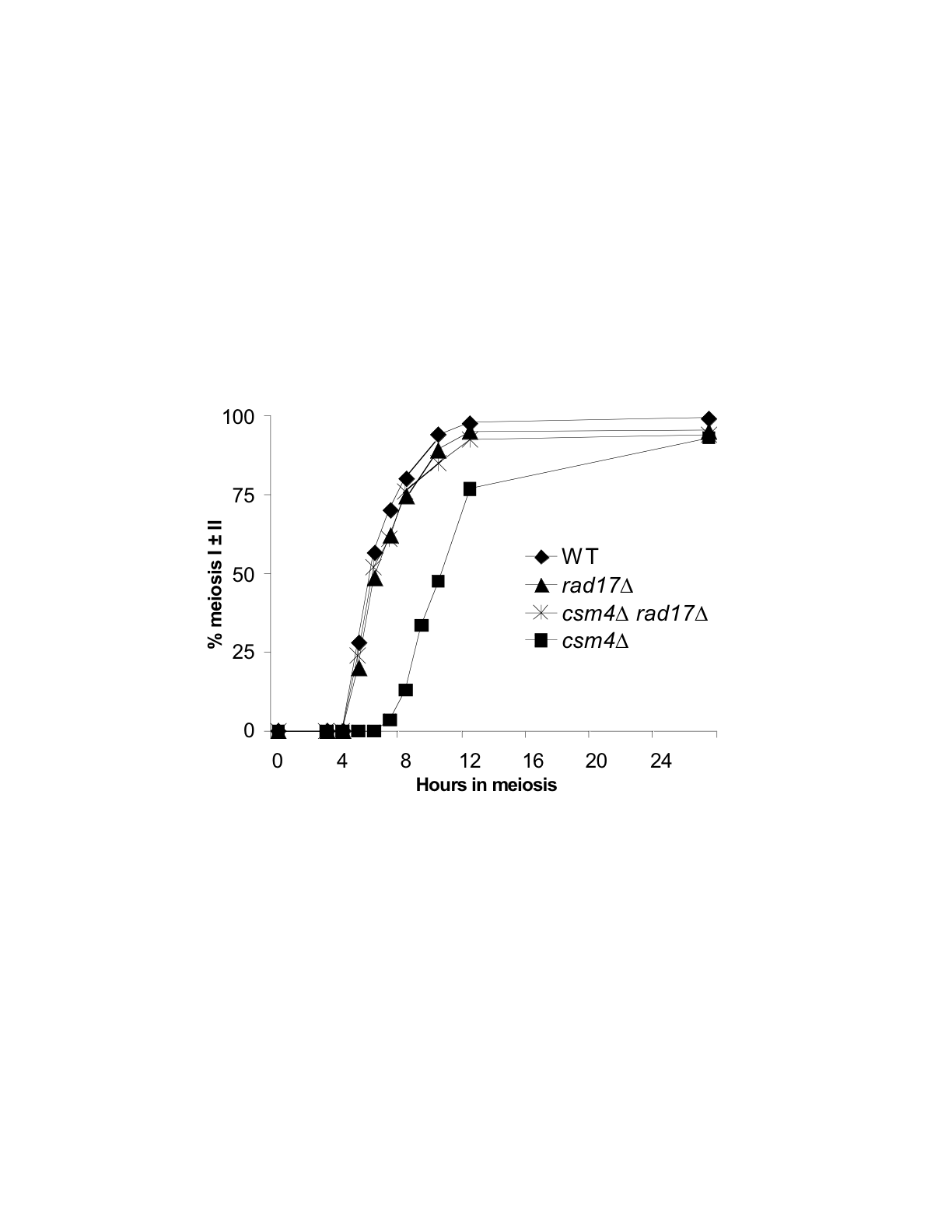

Supplement: Figure S4 — csm4Δ confers a defect in meiotic progression that is suppressed by the rad17Δ mutation. Synchronized meiotic cultures of WT (diamond, EAY1553), csm4Δ (square, EAY1554), rad17Δ (triangle, EAY2201) and csm4Δ rad17Δ (cross, EAY2202) were analyzed for the completion of at least MI (MI+MII) as measured by DAPI staining. A representative experiment is shown. Tetrads dissected from sporulated strains displayed the following percent spore viability: WT-93% (Figure 1), csm4Δ-65% (Figure 1), rad17Δ-17% (175 tetrads dissected), and csm4Δ rad17Δ-1.1% (87 tetrads dissected). (0.07 MB TIF) [file pgen.1000188.s004.tif]
