# Supplementary material for: Csm4, in Collaboration with Ndj1, Mediates Telomere-Led Chromosome Dynamics and Recombination during Yeast Meiosis
Source: PLoS Genet. 2008 Sep 26;4(9):e1000188. doi: 10.1371/journal.pgen.1000188 (PMC2533701; doi:10.1371/journal.pgen.1000188)
Supplement: Table S1 — Strains used in this study. (0.1 MB DOC) [file pgen.1000188.s005.doc]

**Table S1. Strains used in this study.**

| Strains described in Figures 1, 3, 4, 5, S4, and Tables 1, 2, 3, S2, S3, S4. | | | | | | | | | | | | | |
| --- | --- | --- | --- | --- | --- | --- | --- | --- | --- | --- | --- | --- | --- |
|  |  |  |  | |  |  |  |  |  |  |  |  |  |
| EAY1108 |  | *MAT*a*, ho::hisG, lys2, ura3, leu2::hisG, trp1::hisG, URA3-cenXV, iLEU2-chXV, iLYS2-chXV* | | | | | | | | | | | |
|  |  |  |  | |  |  |  |  |  |  |  |  |  |
| EAY1112 |  | *MATα, ho::hisG, lys2, ura3, leu2::hisG, trp1::hisG, ade2::hisG, his3::hisG, iTRP1-cenXV* | | | | | | | | | | | |
|  |  |  |  | |  |  |  |  |  |  |  |  |  |
| ^a^Strains isogenic to EAY1108/EAY1112 but homozygous for: | | | | | | | | | | | | | |
| EAY1418 | / | EAY1419 | | | |  | *csm4Δ::KANMX4* | | | | | | |
| EAY1488 | / | EAY1500 | | | |  | *ndj1Δ::NATMX4* | | | | | | |
| EAY1271 | / | EAY1276 | | | |  | *mlh1Δ::HPHMX4* | | | | | | |
| EAY1281 | / | EAY1279 | | | |  | *msh5Δ::NATMX4* | | | | | | |
| EAY1281 | / | EAY1280 | | | |  | *msh5Δ::NATMX4* | | | | | | |
| EAY1486 | / | EAY1487 | | | |  | *csm4Δ::KANMX4, ndj1Δ::NATMX4* | | | | | | |
| EAY1421 | / | EAY1423 | | | |  | *csm4Δ::KANMX4, mlh1Δ::HPHMX4* | | | | | | |
| EAY1422 | / | EAY1424 | | | |  | *csm4Δ::KANMX4, mlh1Δ::HPHMX4* | | | | | | |
| EAY1136 | / | EAY1135 | | | |  | *csm4Δ::KANMX4, msh5Δ::NATMX4* | | | | | | |
| EAY1979 | / | EAY1980 | | | |  | *rad17Δ::HYGMX4* | | | | | | |
| EAY1981 | / | EAY1982 | | | |  | *csm4Δ::KANMX4 rad17Δ::HPHMX4* | | | | | | |
|  |  |  |  | |  |  |  |  |  |  |  |  |  |
| EAY1553 |  | EAY1108 x EAY1112 | | | | | | | | | | | |
| EAY1554 |  | EAY1418 x EAY1419 | | | | | | | | | | | |
| EAY2201 |  | EAY1979 x EAY1980 | | | | | | | | | | | |
| EAY2202 |  | EAY1981 x EAY1982 | | | | | | | | | | | |
|  |  |  |  | |  |  |  |  |  |  |  |  |  |
| ^b^NH942 |  | *MATα, ho::hisG, ade2Δ, can1, ura3(ΔSma-Pst), met13-B, trp5-S, iURA3-cenVIII, thr1-A, cup1^s^* | | | | | | | | | | | |
| ^b^NH943 |  | *MAT*a*, ho::hisG, ade2Δ, ura3(ΔSma-Pst), leu2::hisG, iADE2-cenIII, lys5-P, his4-B, cyh2* | | | | | | | | | | | |
|  |  |  |  | |  |  |  |  |  |  |  |  |  |
| ^c^Strains isogenic to NH942/NH943 but homozygous for: | | | | | | | | | | | | | |
| EAY1483 | / | EAY1484 | | | | *csm4Δ::KANMX4* | | | | | | | |
|  |  |  |  | |  |  |  |  |  |  |  |  |  |
| ^d^BR4635-8Bα | | | | *MATα, HIS4, leu2::CUP1, arg4-8, iTHR1, iURA3-cenIII, iNAT, iLEU2, iADE2, trp1* | | | | | | | | | |
| ^d^BR4256-5Ba | | | | *MAT*a*, his4-260, leu2:CUP1, arg4-8, iHYG-cenIII, trp1* | | | | | | | | | |
|  |  |  |  | |  |  |  |  |  |  |  |  |  |
| ^e^Strains isogenic to BR4635-8Bα/BR4256-5Ba but homozygous for: | | | | | | | | | | | | | |
| EAY1867 | / | EAY1869 | | | | *csm4Δ::KANMX4* | | | | | | | |
|  |  |  |  | |  |  |  |  |  |  |  |  |  |
| EAY1871 |  | EAY1867 x EAY1869 | | | | | | | | | | | |
| EAY1873 |  | BR4635-8Bα x BR4256-5Ba | | | | | | | | | | | |
|  |  |  |  | |  |  |  |  |  |  |  |  |  |
| Strains described in Figures 2, 8, and S1. | | | | | | | | | | | | | |
|  |  |  |  | |  |  |  |  |  |  |  |  |  |
| NKY3834 | | *MAT*a*/MATα, ho::hisG/", ZIP1::ZIP1-GFP(700)/", ura3(ΔSmaI-PstI::hisG)/", leu2::hisG/", HIS4::LEU2-(Bam)/his4X::LEU2(NgoMIV)-URA3* | | | | | | | | | | | |
|  |  |
| NKY3837 | | *MAT*a*/MATα, ho::hisG/", ZIP1::ZIP1-GFP(700)/", leu2::hisG/", ura3/", ndj1Δ::HPHMX4/", his4::LEU2-URA3/"* | | | | | | | | | | | |
|  |  |
| NKY3906 | | *MAT*a*/MATα, ho::hisG or ho::LYS2/”, ndj1::HPHMX4/”, RAP1-GFP(SVM)-KANMX6/”, HIS4::LEU2(NewBam)* | | | | | | | | | | | |
|  |  |

**Table S1 (continued)**

| NKY3907 | | *MAT*a*/MATα, ho::hisG/”, HIS4-LEU2/HIS4, leu2::hisG/”, spo11(Y153F)::HPHMX4/”, ura3(ΔSmaI-PstI)/”, ndj1Δ::NATMX4/”* | | | | | | | | | | | |
| --- | --- | --- | --- | --- | --- | --- | --- | --- | --- | --- | --- | --- | --- |
|  |  | |  | | | | | | | | | | |
| NKY3908 | | *MAT*a*/MATα, ho::hisG/”, lys2::hisG/LYS2, HIS4-LEU2/HIS4, leu2::hisG/”, spo11(Y153F)::HPHMX4/”, ura3(ΔSmaI-PstI)/”, csm4Δ::HPHMX4/”* | | | | | | | | | | | |
|  |  | |
| NKY4002 | | *MAT*a*/MATα, ho::hisG/”, ZIP1::ZIP1-GFP(700)/ZIP1, csm4Δ::HPHMX4/”, HIS4-LEU2(BamHI, ori)/his4* | | | | | | | | | | | |
|  |  | |
| NKY4000 | | *MAT*a*/MATα, ho::hisG/”, leu2::hisG/”, ura3(ΔSmaI-PstI)/”, HIS4-LEU2(NewBam)/his4X-LEU2-URA3, RAP1-GFP(SVM)-KANMX6/RAP1-GFP(SVM)-KANMX6, SPC42/SPC42-yECFP-KANMX6* | | | | | | | | | | | |
|  |  | |
|  |  | |  |  |  |  |  |  |  |  |  |  |  |
| Strains isogenic to NKY4000, but homozygous for: | | | | | | | | | | | | | |
| NKY3904 | | *csm4Δ::HPHMX4/”* | | | | | | | | | | | |
| NKY3905 | | *csm4Δ::HPHMX4/”, ndj1Δ::NATMX4/”* | | | | | | | | | | | |
|  | |  | | | | | | | | | | | |
| NKY4003 | | *MAT*a*/MATα, ho::hisG/”, ZIP1::ZIP1-GFP(700)/ZIP1, csm4Δ::HPHMX4/”, ndj1Δ::NATMX4/”, HIS4-LEU2(BamHI, ori)/his4* | | | | | | | | | | | |
|  |  | |
| NKY4005 | | *MAT*a*/MATα, ho::hisG/”, HIS4-LEU2(NewBam)/his4X-LEU2-URA3, leu2::hisG/", RAP1-mRFP-HPHMX/”, ura3(ΔSmaI-PstI)/”* | | | | | | | | | | | |
|  |  | |
| EAY1797 | | *MAT*a*/MATα, ho::hisG/”, leu2::hisG/”, ura3(ΔSmaI-PstI)/”, his4X::LEU2/”, RAP1-RFP::HPHMX/”, GFP-CSM4::KANMX4/”* | | | | | | | | | | | |
|  |  | |
|  |  | |  |  |  |  |  |  |  |  |  |  |  |
| Strains described in Figures 6, 7, S2, and S3. | | | | | | | | | | | | | |
|  |  | |  |  |  |  |  |  |  |  |  |  |  |
| NKY3890 | | *MATa/MATα, ho::hisG/", leu2/", ura3/", nuc1::HPHMX4/", HIS4::LEU2-(BamHI)/his4-x::LEU2-(NgoMIV)-URA3* | | | | | | | | | | | |
|  |  | |
| NKY3891 | | *MAT*a*/MATα, ho::hisG/", leu2/", ura3/", nuc1::HPHMX4/", HIS4::LEU2-(BamHI)/his4-x::LEU2-(NgoMIV)-URA3, ndj1Δ::NATMX4/"* | | | | | | | | | | | |
|  |  | |
| NKY3892 | | *MAT*a*/MATα, ho::hisG/", leu2/", ura3/", nuc1::HPHMX4/", HIS4::LEU2-(BamHI)/his4-x::LEU2-(NgoMIV)-URA3, csm4Δ::HPHMX4/"* | | | | | | | | | | | |
|  |  | |
| NKY3893 | | *MAT*a*/MATα, ho::hisG/", leu2/", ura3/", nuc1::HPHMX4/", HIS4::LEU2-(BamHI)/his4-x::LEU2-(NgoMIV)-URA3, ndj1Δ::NATMX4/", csm4Δ::HPHMX4/"* | | | | | | | | | | | |
|  |  | |
| NKY3894 | | *MAT*a*/MATα, ho::hisG/", leu2/", ura3/", HIS4::LEU2-(BamHI)/his4-x::LEU2-(BamHI)-URA3, circular chromosome III/"* | | | | | | | | | | | |
|  |  | |
| NKY3895 | | *MAT*a*/MATα, ho::hisG/", leu2/", ura3/", HIS4::LEU2-(BamHI)/his4-x::LEU2-(BamHI)-URA3, circular chromosome III/", ndj1Δ::NATMX4/"* | | | | | | | | | | | |
|  |  | |
| NKY3896 | | *MAT*a*/MATα, ho::hisG/", leu2/", ura3/",HIS4::LEU2-(BamHI)/his4-x::LEU2-(BamHI)-URA3, circular chromosome III/", csm4Δ::HPHMX4/"* | | | | | | | | | | | |
|  |  | |
| NKY3897 | | *MAT*a*/MATα, ho::hisG/", leu2/", ura3/", HIS4::LEU2-(BamHI)/his4-x::LEU2-(BamHI)-URA3, circular chromosome III/", ndj1Δ::NATMX4/", csm4Δ::HPHMX4/"* | | | | | | | | | | | |
|  |  | |
| NKY3898 | | *MAT*a*/MATα, ho::hisG/", leu2/", ura3/", nuc1::HygroB/", HIS4::LEU2-(BamHI)/his4-x::LEU2-(NgoMIV)-URA3, rad50-KI81::URA3/"* | | | | | | | | | | | |
|  |  | |
| NKY3899 | | *MAT*a*/MATα, ho::hisG/", leu2/", ura3/", nuc1::HygroB/", HIS4::LEU2-(BamHI)/his4-x::LEU2-(NgoMIV)-URA3, ndj1Δ::NATMX4/", rad50-KI81::URA3/"* | | | | | | | | | | | |
|  |  | |
| NKY3900 | | *MAT*a*/MATα, ho::hisG/", leu2/", ura3/", nuc1::HygroB/", HIS4::LEU2-(BamHI)/his4-x::LEU2-(NgoMIV)-URA3, csm4Δ::HPHMX4/", rad50-KI81::URA3/"* | | | | | | | | | | | |
|  |  | |
| NKY3901 | | *MAT*a*/MATα, ho::hisG/", leu2/", ura3/", nuc1::HygroB/", HIS4::LEU2-(BamHI)/his4-x::LEU2-(NgoMIV)-URA3, ndj1Δ::NATMX4/", csm4Δ::HPHMX4/", rad50-KI81::URA3/"* | | | | | | | | | | | |
|  |  | |

^a^The first strain in each pair is isogenic to EAY1108 and the second is isogenic to EAY1112 [27]. EAY1553, EAY1554, EAY2201, and EAY2202 are stable diploids. ^b^Described in de los Santos et al. [46]. ^c^The first strain in each pair is isogenic to NH942 and the second is isogenic to NH943. ^d^Described in Rockmill et al. [29]. ^e^The first strain in each pair is isogenic to BR4635-8Bα and the second is isogenic to BR4256-5Ba. EAY1871 and EAY1873 are stable diploids. EAY1108/EAY1112 are SK1 congenic strains [27] and BR4635-8Bα/BR4256-5Ba are BR-background strains [29]. All other strains listed are SK1 isogenic strains.
