# Supplementary material for: Csm4, in Collaboration with Ndj1, Mediates Telomere-Led Chromosome Dynamics and Recombination during Yeast Meiosis
Source: PLoS Genet. 2008 Sep 26;4(9):e1000188. doi: 10.1371/journal.pgen.1000188 (PMC2533701; doi:10.1371/journal.pgen.1000188)
Supplement: Table S2 — Genetic map distances (cM) and the distribution of parental and recombinant progeny for the NH942/NH943 strain background in WT and csm4Δ on chromosomes III, VI, and, VIII. (0.1 MB DOC) [file pgen.1000188.s006.doc]

**Table S2. Genetic map distances (cM) and the distribution of parental and recombinant progeny for the NH942 / NH943 strain background in WT and *csm4Δ* on chromosomes III, VI, and, VIII.**

| Chromosome III | Tetrads^a^ | | | | |  | Single spores^b^ | | | |
| --- | --- | --- | --- | --- | --- | --- | --- | --- | --- | --- |
| Relevant genotype | Number | cM | PD | TT | NPD |  | Number | cM | Parental | Recombinant |
|  | analyzed |  |  |  |  |  | analyzed |  |  |  |
| *HIS4-LEU2*: |  |  |  |  |  |  |  |  |  |  |
| wild-type | 476 | 12.8 - 15.4 | 352 | 122 | 2 |  | 2350 | 11.7 - 14.5 | 2043 | 307 |
| *csm4Δ* | 530 | 11.2 - 13.8 | 413 | 114 | 3 |  | 3300 | 11.0 - 13.3 | 2900 | 400 |
|  |  |  |  |  |  |  |  |  |  |  |
| *LEU2-ADE2*: |  |  |  |  |  |  |  |  |  |  |
| wild-type | 476 | 5.2 - 6.6 | 420 | 56 | 0 |  | 2350 | 5.7 - 7.8 | 2193 | 157 |
| *csm4Δ* | 530 | 10.7 - 13.7 | 426 | 99 | 5 |  | 3300 | 11.6 - 13.9 | 2881 | 419 |
|  |  |  |  |  |  |  |  |  |  |  |
| *ADE2-MAT*: |  |  |  |  |  |  |  |  |  |  |
| wild-type | 476 | 14.0 - 16.4 | 336 | 139 | 1 |  | 2350 | 13.6 - 16.5 | 1998 | 352 |
| *csm4Δ* | 530 | 17.1 - 20.1 | 353 | 173 | 4 |  | 3300 | 17.4 - 20.1 | 2684 | 616 |

| Chromosome VII | Tetrads^a^ | | | | |  | Single spores^b^ | | | |
| --- | --- | --- | --- | --- | --- | --- | --- | --- | --- | --- |
| Relevant genotype | Number | cM | PD | TT | NPD |  | Number | cM | Parental | Recombinant |
|  | analyzed |  |  |  |  |  | analyzed |  |  |  |
| *LYS5-MET13*: |  |  |  |  |  |  |  |  |  |  |
| wild-type | 464 | 19.1 - 22.1 | 288 | 173 | 3 |  | 2350 | 18.8 - 22.1 | 1871 | 479 |
| *csm4Δ* | 523 | 23.6 - 27.0 | 288 | 229 | 6 |  | 3300 | 22.7 - 25.6 | 2505 | 795 |
|  |  |  |  |  |  |  |  |  |  |  |
| *MET13-CYH2*: |  |  |  |  |  |  |  |  |  |  |
| wild-type | 464 | 7.9 - 9.7 | 382 | 82 | 0 |  | 2350 | 8.1 - 10.5 | 2133 | 217 |
| *csm4Δ* | 523 | 14.0 - 16.2 | 370 | 152 | 1 |  | 3300 | 14.5 - 17.1 | 2781 | 519 |
|  |  |  |  |  |  |  |  |  |  |  |
| *CYH2-TRP5*: |  |  |  |  |  |  |  |  |  |  |
| wild-type | 464 | 34.3 - 38.3 | 167 | 289 | 8 |  | 2350 | 31.9 - 35.7 | 1556 | 794 |
| *csm4Δ* | 523 | 46.7 - 52.3 | 150 | 344 | 29 |  | 3300 | 38.3 - 41.7 | 1980 | 1320 |

**Table S2 (continued)**

| Chromosome VIII | Tetrads^a^ | | | | |  | Single spores^b^ | | | |
| --- | --- | --- | --- | --- | --- | --- | --- | --- | --- | --- |
| Relevant genotype | Number | cM | PD | TT | NPD |  | Number | cM | Parental | Recombinant |
|  | analyzed |  |  |  |  |  | analyzed |  |  |  |
| *URA3-THR1*: |  |  |  |  |  |  |  |  |  |  |
| wild-type | 461 | 20.1 - 22.7 | 269 | 191 | 1 |  | 2350 | 20.9 - 24.4 | 1819 | 531 |
| *csm4Δ* | 527 | 23.2 - 26.4 | 291 | 231 | 5 |  | 3300 | 23.5 - 26.4 | 2478 | 822 |
|  |  |  |  |  |  |  |  |  |  |  |
| *THR1-CUP1*: |  |  |  |  |  |  |  |  |  |  |
| wild-type | 461 | 23.7 - 26.1 | 231 | 230 | 0 |  | 2350 | 23.3 - 26.9 | 1761 | 589 |
| *csm4Δ* | 527 | 27.7 - 31.3 | 256 | 263 | 8 |  | 3300 | 25.4 - 28.4 | 2413 | 887 |

All mutants are isogenic derivatives of NH1942 / NH943. ^a^Intervals correspond to the genetic distance calculated from tetrads +/- one standard error. Standard error was calculated using the Stahl Laboratory Online Tools website (http://www.molbio.uoregon.edu/~fstahl/). ^b^Data shown as 95% confidence intervals around the recombination frequency determined from single spores. To facilitate comparisons to the tetrad data, recombination frequencies obtained from single spore data were multiplied by 100 to yield genetic map distances (cM). The recombination frequency in single spores determined by: Parental / (Parental + Recombinant) and cM indicates the genetic distance in tetrads calculated using the formula of Perkins [38]: 50 x {TT + (6 x NPD)} / (PD + TT + NPD).
