# Supplementary material for: Csm4, in Collaboration with Ndj1, Mediates Telomere-Led Chromosome Dynamics and Recombination during Yeast Meiosis
Source: PLoS Genet. 2008 Sep 26;4(9):e1000188. doi: 10.1371/journal.pgen.1000188 (PMC2533701; doi:10.1371/journal.pgen.1000188)
Supplement: Table S3 — Interference as measured by the Malkova method. (0.2 MB DOC) [file pgen.1000188.s007.doc]

**Table S3. Interference as measured by the Malkova method.**

|  |  | ***URA3 - LEU2*** | | | | | | | | | | | | | | | ***LEU2 - LYS2*** | | | | | | | | | | | | | | |
| --- | --- | --- | --- | --- | --- | --- | --- | --- | --- | --- | --- | --- | --- | --- | --- | --- | --- | --- | --- | --- | --- | --- | --- | --- | --- | --- | --- | --- | --- | --- | --- |
| **wild-type** | Interval | *LEU2 - LYS2* | | | | | *LYS2 - ADE2* | | | | | *ADE2 - HIS3* | | | | | *URA3 - LEU2* | | | | | *LYS2 - ADE3* | | | | | *ADE3 - HIS3* | | | | |
|  | PD | 212 | : | 392 | : | 3 | 457 | : | 148 | : | 2 | 180 | : | 415 | : | 12 | 212 | : | 280 | : | 4 | 296 | : | 198 | : | 2 | 157 | : | 331 | : | 8 |
|  | cM | 33.8 | | ± | 1.2 | | 13.2 | | ± | 1.1 | | 40.1 | | ± | 1.8 | | 30.6 | | ± | 1.6 | | 21.2 | | ± | 1.4 | | 38.2 | | ± | 1.8 | |
|  | TT + NPD | 284 | : | 177 | : | 0 | 346 | : | 115 | : | 0 | 163 | : | 294 | : | 4 | 395 | : | 176 | : | 1 | 507 | : | 65 | : | 0 | 186 | : | 378 | : | 8 |
|  | cM | 19.2 | | ± | 1.1 | | 12.5 | | ± | 1 | | 34.5 | | ± | 1.6 | | 15.9 | | ± | 1.1 | | 5.7 | | ± | 0.7 | | 37.2 | | ± | 1.6 | |
|  | *p* | 1.2E-17 | | | | | 0.317 | | | | | 0.056 | | | | | 2.5E-17 | | | | | 1.2E-27 | | | | | 0.923 | | | | |
|  | Significant? | Y | | | | | N | | | | | N | | | | | Y | | | | | Y | | | | | N | | | | |
|  | Ratio | 0.568 | | | | | 0.947 | | | | | 0.860 | | | | | 0.520 | | | | | 0.269 | | | | | 0.974 | | | | |

|  |  | ***URA3 - LEU2*** | | | | | | | | | | | | | | | ***LEU2 - LYS2*** | | | | | | | | | | | | | | |
| --- | --- | --- | --- | --- | --- | --- | --- | --- | --- | --- | --- | --- | --- | --- | --- | --- | --- | --- | --- | --- | --- | --- | --- | --- | --- | --- | --- | --- | --- | --- | --- |
| ***csm4Δ*** | Interval | *LEU2 - LYS2* | | | | | *LYS2 - ADE2* | | | | | *ADE2 - HIS3* | | | | | *URA3 - LEU2* | | | | | *LYS2 - ADE3* | | | | | *ADE3 - HIS3* | | | | |
|  | PD | 64 | : | 137 | : | 2 | 143 | : | 60 | : | 0 | 48 | : | 145 | : | 10 | 64 | : | 146 | : | 6 | 130 | : | 86 | : | 0 | 49 | : | 152 | : | 15 |
|  | cM | 36.7 | | ± | 2.5 | | 14.8 | | ± | 2 | | 50.5 | | ± | 4.3 | | 42.1 | | ± | 3.3 | | 19.9 | | ± | 2 | | 56 | | ± | 4.7 | |
|  | TT + NPD | 152 | : | 175 | : | 1 | 219 | : | 108 | : | 1 | 72 | : | 233 | : | 23 | 139 | : | 173 | : | 3 | 232 | : | 82 | : | 1 | 71 | : | 226 | : | 18 |
|  | cM | 27.6 | | ± | 1.6 | | 17.4 | | ± | 2 | | 56.6 | | ± | 3.9 | | 30.3 | | ± | 2 | | 14 | | ± | 2 | | 53 | | ± | 3.6 | |
|  | *p* | 0.002 | | | | | 0.435 | | | | | 0.586 | | | | | 0.001 | | | | | 0.002 | | | | | 0.842 | | | | |
|  | Significant? | Y | | | | | N | | | | | N | | | | | Y | | | | | Y | | | | | N | | | | |
|  | Ratio | 0.752 | | | | | 1.176 | | | | | 1.121 | | | | | 0.720 | | | | | 0.704 | | | | | 0.946 | | | | |

|  |  | ***URA3 - LEU2*** | | | | | | | | | | | | | | | ***LEU2 - LYS2*** | | | | | | | | | | | | | | |
| --- | --- | --- | --- | --- | --- | --- | --- | --- | --- | --- | --- | --- | --- | --- | --- | --- | --- | --- | --- | --- | --- | --- | --- | --- | --- | --- | --- | --- | --- | --- | --- |
| ***ndj1Δ*** | Interval | *LEU2 - LYS2* | | | | | *LYS2 - ADE2* | | | | | *ADE2 - HIS3* | | | | | *URA3 - LEU2* | | | | | *LYS2 - ADE3* | | | | | *ADE3 - HIS3* | | | | |
|  | PD | 59 | : | 113 | : | 1 | 115 | : | 58 | : | 0 | 42 | : | 117 | : | 14 | 59 | : | 126 | : | 7 | 91 | : | 101 | : | 0 | 46 | : | 133 | : | 13 |
|  | cM | 34.4 | | ± | 2.4 | | 16.8 | | ± | 2 | | 58.1 | | ± | 5.7 | | 43.8 | | ± | 4 | | 26.3 | | ± | 2 | | 54.9 | | ± | 5 | |
|  | TT + NPD | 133 | : | 161 | : | 5 | 179 | : | 120 | : | 0 | 66 | : | 213 | : | 20 | 114 | : | 163 | : | 3 | 203 | : | 77 | : | 0 | 62 | : | 197 | : | 21 |
|  | cM | 31.9 | | ± | 2.5 | | 20.1 | | ± | 1 | | 55.7 | | ± | 4 | | 32.3 | | ± | 2.2 | | 13.8 | | ± | 1 | | 57.7 | | ± | 4.3 | |
|  | *p* | 0.037 | | | | | 0.358 | | | | | 0.464 | | | | | 2.1E-02 | | | | | 2.4E-07 | | | | | 0.875 | | | | |
|  | Significant? | Y | | | | | N | | | | | N | | | | | Y | | | | | Y | | | | | N | | | | |
|  | Ratio | 0.927 | | | | | 1.196 | | | | | 0.959 | | | | | 0.737 | | | | | 0.525 | | | | | 1.051 | | | | |

|  |  | ***URA3 - LEU2*** | | | | | | | | | | | | | | | ***LEU2 - LYS2*** | | | | | | | | | | | | | | |
| --- | --- | --- | --- | --- | --- | --- | --- | --- | --- | --- | --- | --- | --- | --- | --- | --- | --- | --- | --- | --- | --- | --- | --- | --- | --- | --- | --- | --- | --- | --- | --- |
| ***csm4Δ ndj1Δ*** | Interval | *LEU2 - LYS2* | | | | | *LYS2 - ADE2* | | | | | *ADE2 - HIS3* | | | | | *URA3 - LEU2* | | | | | *LYS2 - ADE3* | | | | | *ADE3 - HIS3* | | | | |
|  | PD | 85 | : | 244 | : | 8 | 219 | : | 114 | : | 4 | 83 | : | 226 | : | 28 | 85 | : | 228 | : | 9 | 176 | : | 142 | : | 4 | 84 | : | 216 | : | 22 |
|  | cM | 43.3 | | ± | 2.5 | | 20.5 | | ± | 2 | | 58.5 | | ± | 4.1 | | 43.8 | | ± | 2.7 | | 25.8 | | ± | 2 | | 54 | | ± | 3.9 | |
|  | TT + NPD | 237 | : | 211 | : | 4 | 295 | : | 153 | : | 4 | 110 | : | 316 | : | 26 | 252 | : | 209 | : | 6 | 338 | : | 125 | : | 4 | 109 | : | 326 | : | 32 |
|  | cM | 26 | | ± | 1.7 | | 19.6 | | ± | 2 | | 52.2 | | ± | 3 | | 26.2 | | ± | 1.8 | | 16 | | ± | 2 | | 55.5 | | ± | 3.2 | |
|  | *p* | 4.2E-14 | | | | | 0.917 | | | | | 0.358 | | | | | 5.2E-14 | | | | | 2.0E-06 | | | | | 0.673 | | | | |
|  | Significant? | Y | | | | | N | | | | | N | | | | | Y | | | | | Y | | | | | N | | | | |
|  | Ratio | 0.600 | | | | | 0.956 | | | | | 0.892 | | | | | 0.598 | | | | | 0.620 | | | | | 1.028 | | | | |

**Table S3 (continued)**

|  |  | ***LYS2 - ADE2*** | | | | | | | | | | | | | | | ***ADE2 - HIS3*** | | | | | | | | | | | | | | |
| --- | --- | --- | --- | --- | --- | --- | --- | --- | --- | --- | --- | --- | --- | --- | --- | --- | --- | --- | --- | --- | --- | --- | --- | --- | --- | --- | --- | --- | --- | --- | --- |
| **wild-type** | Interval | *URA3 - LEU2* | | | | | *LEU2 - LYS2* | | | | | *ADE3 - HIS3* | | | | | *URA3 - LEU2* | | | | | *LEU2 - LYS2* | | | | | *LYS2 - ADE2* | | | | |
|  | PD | 457 | : | 351 | : | 5 | 296 | : | 505 | : | 2 | 236 | : | 556 | : | 11 | 180 | : | 160 | : | 1 | 157 | : | 185 | : | 1 | 236 | : | 107 | : | 0 |
|  | cM | 23.1 | | ± | 1.2 | | 32.2 | | ± | 1 | | 38.7 | | ± | 1.4 | | 24.5 | | ± | 1.6 | | 27.8 | | ± | 2 | | 15.6 | | ± | 1.3 | |
|  | TT + NPD | 150 | : | 115 | : | 0 | 200 | : | 64 | : | 1 | 107 | : | 153 | : | 5 | 427 | : | 294 | : | 4 | 339 | : | 384 | : | 2 | 567 | : | 156 | : | 2 |
|  | cM | 21.7 | | ± | 1.5 | | 13.2 | | ± | 2 | | 34.5 | | ± | 2.7 | | 21.9 | | ± | 1.2 | | 27.3 | | ± | 1 | | 11.6 | | ± | 0.9 | |
|  | *p* | 0.243 | | | | | 1.3E-27 | | | | | 0.003 | | | | | 0.130 | | | | | 0.955 | | | | | 0.002 | | | | |
|  | Significant? | N | | | | | Y | | | | | Y | | | | | N | | | | | N | | | | | Y | | | | |
|  | Ratio | 0.939 | | | | | 0.410 | | | | | 0.891 | | | | | 0.894 | | | | | 0.982 | | | | | 0.744 | | | | |

|  |  | ***LYS2 - ADE2*** | | | | | | | | | | | | | | | ***ADE2 - HIS3*** | | | | | | | | | | | | | | |
| --- | --- | --- | --- | --- | --- | --- | --- | --- | --- | --- | --- | --- | --- | --- | --- | --- | --- | --- | --- | --- | --- | --- | --- | --- | --- | --- | --- | --- | --- | --- | --- |
| ***csm4Δ*** | Interval | *URA3 - LEU2* | | | | | *LEU2 - LYS2* | | | | | *ADE3 - HIS3* | | | | | *URA3 - LEU2* | | | | | *LEU2 - LYS2* | | | | | *LYS2 - ADE2* | | | | |
|  | PD | 145 | : | 214 | : | 6 | 130 | : | 229 | : | 6 | 75 | : | 262 | : | 28 | 48 | : | 71 | : | 1 | 49 | : | 71 | : | 0 | 75 | : | 45 | : | 0 |
|  | cM | 34.2 | | ± | 2.2 | | 36.3 | | ± | 2 | | 58.9 | | ± | 3.8 | | 32.1 | | ± | 3.2 | | 29.6 | | ± | 2 | | 18.8 | | ± | 2.2 | |
|  | TT + NPD | 60 | : | 106 | : | 3 | 86 | : | 83 | : | 0 | 45 | : | 119 | : | 5 | 157 | : | 249 | : | 8 | 167 | : | 241 | : | 6 | 290 | : | 123 | : | 1 |
|  | cM | 36.7 | | ± | 3.3 | | 24.6 | | ± | 2 | | 44.1 | | ± | 3.8 | | 35.9 | | ± | 2.2 | | 33.5 | | ± | 2 | | 15.6 | | ± | 1.3 | |
|  | *p* | 0.645 | | | | | 0.001 | | | | | 0.037 | | | | | 0.638 | | | | | 0.215 | | | | | 0.221 | | | | |
|  | Significant? | N | | | | | Y | | | | | Y | | | | | N | | | | | N | | | | | N | | | | |
|  | Ratio | 1.073 | | | | | 0.678 | | | | | 0.749 | | | | | 1.118 | | | | | 1.132 | | | | | 0.830 | | | | |

|  |  | ***LYS2 - ADE2*** | | | | | | | | | | | | | | | ***ADE2 - HIS3*** | | | | | | | | | | | | | | |
| --- | --- | --- | --- | --- | --- | --- | --- | --- | --- | --- | --- | --- | --- | --- | --- | --- | --- | --- | --- | --- | --- | --- | --- | --- | --- | --- | --- | --- | --- | --- | --- |
| ***ndj1Δ*** | Interval | *URA3 - LEU2* | | | | | *LEU2 - LYS2* | | | | | *ADE3 - HIS3* | | | | | *URA3 - LEU2* | | | | | *LEU2 - LYS2* | | | | | *LYS2 - ADE2* | | | | |
|  | PD | 115 | : | 172 | : | 7 | 91 | : | 197 | : | 6 | 67 | : | 200 | : | 27 | 42 | : | 66 | : | 0 | 46 | : | 60 | : | 2 | 67 | : | 41 | : | 0 |
|  | cM | 36.4 | | ± | 2.8 | | 39.6 | | ± | 3 | | 61.6 | | ± | 4.6 | | 30.6 | | ± | 2.3 | | 33.3 | | ± | 4 | | 19 | | ± | 2.3 | |
|  | TT + NPD | 58 | : | 117 | : | 3 | 101 | : | 77 | : | 0 | 41 | : | 130 | : | 7 | 131 | : | 223 | : | 10 | 146 | : | 214 | : | 4 | 227 | : | 137 | : | 0 |
|  | cM | 37.9 | | ± | 3.1 | | 21.6 | | ± | 2 | | 48.3 | | ± | 4.1 | | 38.9 | | ± | 2.6 | | 32.7 | | ± | 2 | | 18.8 | | ± | 1.3 | |
|  | *p* | 0.285 | | | | | 3.4E-08 | | | | | 0.080 | | | | | 0.068 | | | | | 0.735 | | | | | 0.998 | | | | |
|  | Significant? | N | | | | | Y | | | | | N | | | | | N | | | | | N | | | | | N | | | | |
|  | Ratio | 1.041 | | | | | 0.545 | | | | | 0.784 | | | | | 1.271 | | | | | 0.982 | | | | | 0.989 | | | | |

|  |  | ***LYS2 - ADE2*** | | | | | | | | | | | | | | | ***ADE2 - HIS3*** | | | | | | | | | | | | | | |
| --- | --- | --- | --- | --- | --- | --- | --- | --- | --- | --- | --- | --- | --- | --- | --- | --- | --- | --- | --- | --- | --- | --- | --- | --- | --- | --- | --- | --- | --- | --- | --- |
| ***csm4Δ ndj1Δ*** | Interval | *URA3 - LEU2* | | | | | *LEU2 - LYS2* | | | | | *ADE3 - HIS3* | | | | | *URA3 - LEU2* | | | | | *LEU2 - LYS2* | | | | | *LYS2 - ADE2* | | | | |
|  | PD | 219 | : | 286 | : | 9 | 176 | : | 329 | : | 9 | 119 | : | 348 | : | 47 | 83 | : | 105 | : | 5 | 84 | : | 105 | : | 4 | 119 | : | 72 | : | 2 |
|  | cM | 33.1 | | ± | 1.9 | | 37.3 | | ± | 2 | | 61.3 | | ± | 3.5 | | 35 | | ± | 3.6 | | 33.4 | | ± | 3 | | 21.8 | | ± | 2.7 | |
|  | TT + NPD | 118 | : | 151 | : | 6 | 146 | : | 126 | : | 3 | 74 | : | 194 | : | 7 | 254 | : | 332 | : | 10 | 238 | : | 350 | : | 8 | 395 | : | 195 | : | 6 |
|  | cM | 34 | | ± | 2.8 | | 26.2 | | ± | 2 | | 42.9 | | ± | 2.8 | | 32.9 | | ± | 1.7 | | 33.4 | | ± | 2 | | 19.4 | | ± | 1.5 | |
|  | *p* | 0.908 | | | | | 2.0E-06 | | | | | 0.001 | | | | | 0.724 | | | | | 0.494 | | | | | 0.504 | | | | |
|  | Significant? | N | | | | | Y | | | | | Y | | | | | N | | | | | N | | | | | N | | | | |
|  | Ratio | 1.027 | | | | | 0.702 | | | | | 0.700 | | | | | 0.940 | | | | | 1.000 | | | | | 0.890 | | | | |

Crossover interference was analyzed as described by Malkova et al. [37] and Martini et al. [34] for chromosome XV in the EAY1108 / EAY1112 strain background. For each genetic interval, tetrads were divided into two groups based on the presence (NPD, TT) or absence (PD) of a recombination event. For each group, the map distance was measured in the adjacent interval, thus obtaining two map distances for that interval. Differences of tetrad distributions with p < 0.05 were considered significant evidence of interference as assessed by a G test. Map distances (cM) with 95% confidence intervals were calculated as described previously. Like Martini et al. [34], we also present the data as a ratio of the two map distances, with a smaller ratio indicating stronger interference.
