# Supplementary material for: Csm4, in Collaboration with Ndj1, Mediates Telomere-Led Chromosome Dynamics and Recombination during Yeast Meiosis
Source: PLoS Genet. 2008 Sep 26;4(9):e1000188. doi: 10.1371/journal.pgen.1000188 (PMC2533701; doi:10.1371/journal.pgen.1000188)
Supplement: Table S4 — Crossing over in WT tetrads, csm4Δ tetrads, and csm4Δ disomic spores. (0.03 MB DOC) [file pgen.1000188.s008.doc]

**Table S4. Crossing over in WT tetrads, *csm4Δ* tetrads, and *csm4Δ* disomic spores.**

| Genotype: |  | wild-type | | |  | *csm4Δ* | | |  | *csm4Δ* disomes | | |
| --- | --- | --- | --- | --- | --- | --- | --- | --- | --- | --- | --- | --- |
|  |  | Parental | Recombinant | cM |  | Parental | Recombinant | cM |  | Parental | Recombinant^g^ | cM |
| *HIS4-iTHR1*^a^ |  | 912 | 268 | 20.4 - 25.2 |  | 564 | 160 | 19.2 - 25.3 |  | 163 | 22 | 7.8 - 17.7 |
| *iTHR1-iURA3*^b^ |  | 1140 | 40 | 2.5 - 4.6 |  | 710 | 14 | 1.1 - 3.3 |  | 183 | 2 | 0.2 - 4.3 |
| *iURA3-iNAT*^c^ |  | 1180 | 0 | 0 - 0.4 |  | 714 | 10 | 0.7 - 2.6 |  | 181 | 4 | 0.7 - 5.8 |
| *iNAT-iLEU2*^d^ |  | 1154 | 26 | 1.5 - 3.3 |  | 710 | 14 | 1.1 - 3.3 |  | 155 | 30 | 11.4 - 22.5 |
| *iLEU2-MAT*^e^ |  | 954 | 226 | 17.0 - 21.5 |  | 578 | 146 | 17.3 - 23.3 |  | 133 | 52 | 21.9 - 35.3 |
| *MAT-iADE*^f^ |  | 826 | 354 | 27.4 - 32.7 |  | 556 | 168 | 20.2 - 26.5 |  | 137 | 48 | 19.9 - 33.0 |

95% confidence intervals around the recombination frequency were calculated using the VassarStats website (http://faculty.vassar.edu/lowry/VassarStats.html). Crossing over was measured in the six intervals shown for chromosome III as described in the Materials and Methods. “i” indicates insertion at an ectopic location. ^a^Disomes that require Histidine but not Threonine for growth (or require Threonine but not Histidine for growth) represent COs in the *HIS4-iTHR1* interval. ^b^Disomes that are auxotrophic for Threonine represent COs in the *iTHR1-iURA3* interval. ^c^Disomes that are sensitive to nourseothricin represent COs in the *iURA3-iNAT* interval. ^d^Disomes that are resistant to nourseothricin and are Leucine auxotrophs (or sensitive to nourseothricin and are Leucine prototrophs) represent COs in the *iNAT-iLEU2* interval. ^e^Disomes that are Leucine prototrophs and able to mate (or Leucine auxotrophs and non-maters) represent COs in the *iLEU2-MAT* interval. ^f^Disomes that are unable to mate and are Adenine auxotrophs (or able to mate and are Adenine prototrophs) represent COs in the *MAT-iADE2* interval. ^g^To calculate genetic map distances in disomes, the number of recombinants were multiplied by two in order to account for the inability to detect disomes homozygous for dominant markers. Recombination frequencies obtained from single spore and disome data were multiplied by 100 to yield genetic map distances (cM).
